# Prediction of Lung Cancer Metastasis Risk Based on Single‐Cell Metabolic Profiling of Circulating Tumor Cells

**DOI:** 10.1002/advs.202508878

**Published:** 2025-07-18

**Authors:** Yang Xu, Xuesen Hu, Yuan Yuan, Wenwen Liu, Jian Wang, Chunhui Yang, Xianzhe Shi, Wangshu Qin, Liliang Wen, Manqing Lin, Yinuo Jin, Wei Wang, Chunxiu Hu, Guowang Xu, Qi Wang

**Affiliations:** ^1^ Department of Respiratory Medicine Cancer Translational Medicine Research Center The Second Affiliated Hospital of Dalian Medical University Dalian Liaoning 116023 P. R. China; ^2^ State Key Laboratory of Medical Proteomics Dalian Institute of Chemical Physics Chinese Academy of Sciences (CAS) University of Chinese Academy of Sciences Liaoning Province Key Laboratory of Metabolomics Dalian Liaoning 116023 P. R. China; ^3^ Cancer Translational Medicine Research Center The Second Affiliated Hospital of Dalian Medical University Medical College of Tarim University Xinjiang Uygur Autonomous Region Aral 843300 P. R. China; ^4^ School of Integrated Circuits Peking University Beijing 100871 P. R. China

**Keywords:** circulating tumor cells, lung cancer metastasis, metabolic profiling, personalized clinical management, single‐cell analysis

## Abstract

Lung cancer metastasis is a leading cause of cancer‐related mortality, necessitating innovative approaches for early prediction and personalized clinical management. A novel strategy is present to predict lung cancer metastasis risk by combining single‐cell metabolic profiling of circulating tumor cells (CTCs) with a self‐developed CTC sorting and capture platform, enabling high‐efficiency, high‐viability CTC isolation from blood. Using nanoelectrospray ionization‐atmospheric pressure chemical ionization mass spectrometry, single‐cell metabolomic profiling on 301 CTCs derived from patients and animal models are performed. 390 unique metabolites are identified and discovered distinct metabolic signatures associated with different metastatic potentials (brain and bone). Based on these metabolic profiles, a classification model that categorizes CTCs into subgroups with distinct metastatic risks are constructed. The model outperformed traditional clinical indicators and total CTC counts, achieving AUCs of 0.74 (brain metastasis) and 0.92 (bone metastasis). Prospective validation confirmed its metabolite‐based classification accuracy for one‐year metastasis risk prediction. This study highlights the potential of single‐cell metabolomics to uncover novel therapeutic targets and prognostic markers, advancing liquid biopsy from quantitative counting to qualitative analysis. The approach represents a significant advancement in precision medicine for lung cancer management, offering a personalized strategy for predicting metastasis risk and guiding clinical treatment.

## Graphical abstract

A self‐developed CTC sorting and capture platform enabled single‐cell metabolomics of patient/animal CTCs, revealing brain/bone metastasis‐specific metabolic signatures. A classification model developed from these signatures predicted lung cancer metastasis risk, outperforming traditional markers and total CTC counts (AUCs:89 brain = 0.74; bone = 0.92). Prospective validation confirmed its 1‐year prediction accuracy.

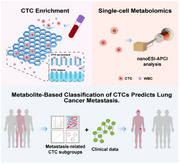

## 1 Introduction

Lung cancer is a major global health burden, causing a large number of cancer‐related deaths each year.^[^ ^1^ ^]^ The high metastatic tendency of lung cancer is a crucial factor influencing its prognosis, as distant metastasis is the main obstacle to long‐term survival.^[^ ^2^ ^]^ Despite advancements in imaging and histopathological diagnostics, early detection of metastatic propensity remains challenging. Current methods such as positron emission tomography (PET) and computed tomography (CT) lack the sensitivity to predict metastasis before clinical symptoms appear. This highlights the urgent need for noninvasive biomarkers to identify high‐risk patients and guiding personized treatments.^[^ ^3^ ^]^

Circulating tumor cells (CTCs), which detach from primary tumors and circulate in the bloodstream, are key drivers of metastasis and hold promise for liquid biopsy.^[^ ^4^ ^]^ The presence and count of CTCs have been clinically linked to poor prognosis^[^ ^5^, ^6^ ^]^ and higher metastasis risk^[^ ^7^ ^]^ in various cancers, particularly in lung cancer.^[^ ^8^ ^]^ However, its clinical utility is limited by CTC heterogeneity and the inability to distinguish subpopulations with different metastatic potentials.^[^ ^9^ ^]^ Conventional CTC detection methods like epithelial cell adhesion molecules (EpCAM)‐based enrichment, preferentially capture epithelial‐like CTCs while neglecting mesenchymal phenotypes,^[^ ^10^ ^]^ which are vital for metastasis.^[^ ^11^ ^]^ Moreover, existing platform often compromise cell viability during enrichment,^[^ ^12^, ^13^ ^]^ limiting downstream functional and molecular analyses.^[^ ^14^ ^]^ These challenges emphasize the need for technologies that ensure both high capture efficiency and cellular integrity.^[^ ^15^, ^16^ ^]^

Metabolism plays a pivotal role in cancer progression and metastasis,^[^ ^17^, ^18^ ^]^ with metabolic reprogramming being a hallmark of metastatic cells.^[^ ^19^, ^20^ ^]^ During metastasis, CTCs undergo metabolic alterations that enable them to survive in the circulation and eventually colonize distant organs.^[^ ^21^, ^22^ ^]^ This metabolic plasticity is a key factor underlying the differential metastatic potential observed among CTCs.^[^ ^23^ ^]^ Therefore, understanding the metabolic profile of individual CTCs could provide insights into their metastatic capabilities and offer a novel approach to stratify patients based on their risk of developing metastasis. Traditional bulk metabolomics masks cellular heterogeneity, failing to resolve metabolic variations in individual CTCs.^[^ ^24^ ^]^ Single‐cell metabolomics (SCM) has recently emerged as a powerful tool to resolve this metabolic heterogeneity at a high resolution. For example, Abouleila et al. utilized live single‐cell mass spectrometry (LSC‐MS) combined with a microfluidics‐based enrichment technique to identify unique metabolic signatures that differentiate CTCs from lymphocytes and distinguish CTCs from different cancer types, highlighting the potential of CTC metabolomics for cancer diagnosis and treatment.^[^ ^25^ ^]^ Neumann et al. employed high‐resolution MS (HRMS) to analyze lipid heterogeneity across over 30 000 individual rat cerebellar cells, demonstrating the power of single‐cell MS to uncover lipid diversity and cellular heterogeneity without relying on bulk sampling.^[^ ^26^ ^]^ Zhang et al. employed integrated multiomic analysis to uncover amino acid dysregulation in hepatocellular carcinoma cells at single‐cell resolution, emphasizing how single‐cell technologies can decode tumor‐immune dynamics for personalized therapies.^[^ ^27^ ^]^ However, three critical challenges still remain in translating SCM into clinically applicable CTC analysis tools: First, current methods (*e.g*., laser capture microdissection^[^ ^28^ ^]^ or microfluidic isolation^[^ ^29^ ^]^) require cell fixation or lysis, which disrupts real‐time metabolic dynamics. Second, the rarity of CTCs (1‐100 cells/mL blood) and low metabolite abundance together with transient metabolite instability demand platforms with high single‐cell capture efficiency (>90%), analytical sensitivity (at the amol level) and clinical sample compatibility. Most critically, most SCM studies currently rely on cultural cell lines or mouse models, leaving significant gaps in the metabolomic databases of clinically derived CTCs. In particular, specific metabolic biomarkers for CTCs with different metastatic organs have not yet been systematically established.

To address these gaps, we proposed three key questions: 1) Can a CTC enrichment platform achieve high capture efficiency while preserving metabolic activity for single‐cell analysis? 2) Does single‐cell metabolomic heterogeneity distinguish CTC subsets with distinct metastatic potentials? 3) Can metabolic signatures predict lung cancer metastasis risk more accurately than conventional biomarkers?

Herein, we present a self‐developed CTC sorting and capture platform that integrates negative enrichment with a Parylene C micropore‐arrayed membrane, optimized for minimal cell damage (viability > 87%) and high recovery rates (>89%). Coupled with nanoelectrospray ionization‐atmospheric pressure chemical ionization (nanoESI‐APCI) MS, we performed single‐cell metabolomic profiling of CTCs from lung cancer patients and metastatic variant cell lines (bone metastasis [Bom] and brain metastasis [Brm]). Our platform's non‐destructive release mechanism, enabled by a polyvinyl alcohol (PVA)‐coated membrane, ensures cell viability and compatibility with downstream culture—a significant improvement over existing technologies.

**Figure 1:**
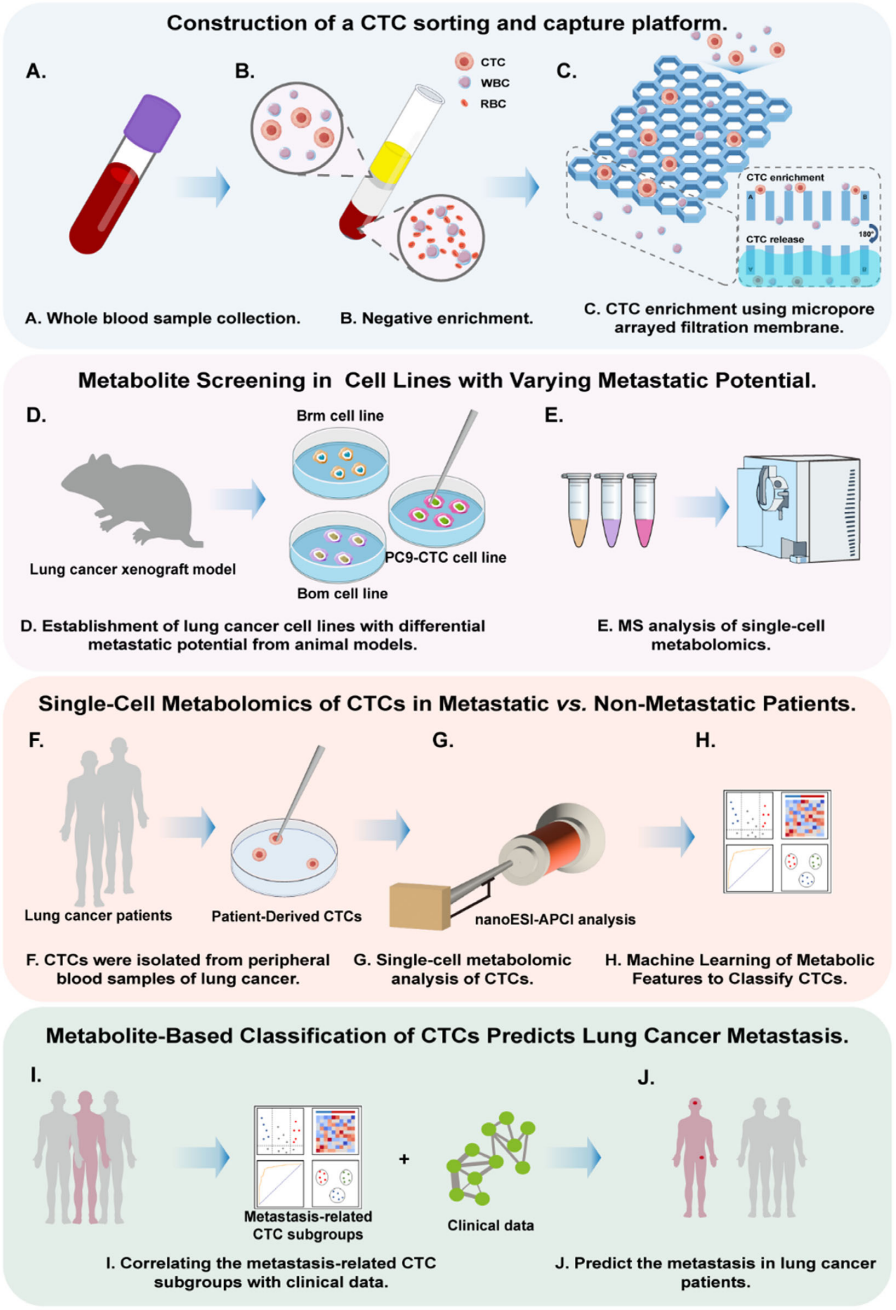
Schematic workflow of predicting lung cancer distant metastasis using a self‐developed CTC sorting and capture platform coupled with single‐cell MS metabolomic analysis. First, individual CTCs were efficiently isolated from whole blood samples obtained from lung cancer patients and animal models. Next, single‐cell metabolomic profiling was performed using nanoelectrospray ionization‐atmospheric pressure chemical ionization (nanoESI‐APCI). Subsequently, metabolite screening was conducted in human lung cancer cell lines with distinct metastatic potentials, including Brm variant cells, Bom variant cells, and the PC9‐CTC cells, to identify differentially abundant metabolites. Finally, the identified metastasis‐associated metabolites were used to classify CTC subgroups. These clinically relevant CTC subgroups with metastatic potential were correlated with clinical data to develop a predictive classification system for lung cancer metastasis.

By analyzing 181 single cells, we identified 390 metabolites and uncovered pathway‐specific differences: Bom cells exhibited enriched arginine biosynthesis, while Brm cells showed elevated phenylalanine metabolism. These findings were translated into a metabolite‐based classification model, stratifying CTCs into three subgroups (CTC1, CTC2, and CTC3). Prospective clinical validation revealed CTC1 and CTC2 as robust predictors of brain and bone metastasis, respectively, outperforming total CTC counts and traditional biomarkers (AUC: 0.74–0.92). Our findings indicate that metabolic heterogeneity within individual CTCs can predict the metastatic potential of lung cancer, enabling more personalized clinical management. Moreover, the metabolic markers identified may serve as potential therapeutic targets^[^ ^30^ ^]^ and enhance our understanding of cancer metastasis (**Figure** **1**).^[^ ^31^ ^]^

## 2 Results and Discussion

### 2.1 Design and Performance Evaluation of the CTC Sorting and Capture Platform

**Figure 2:**
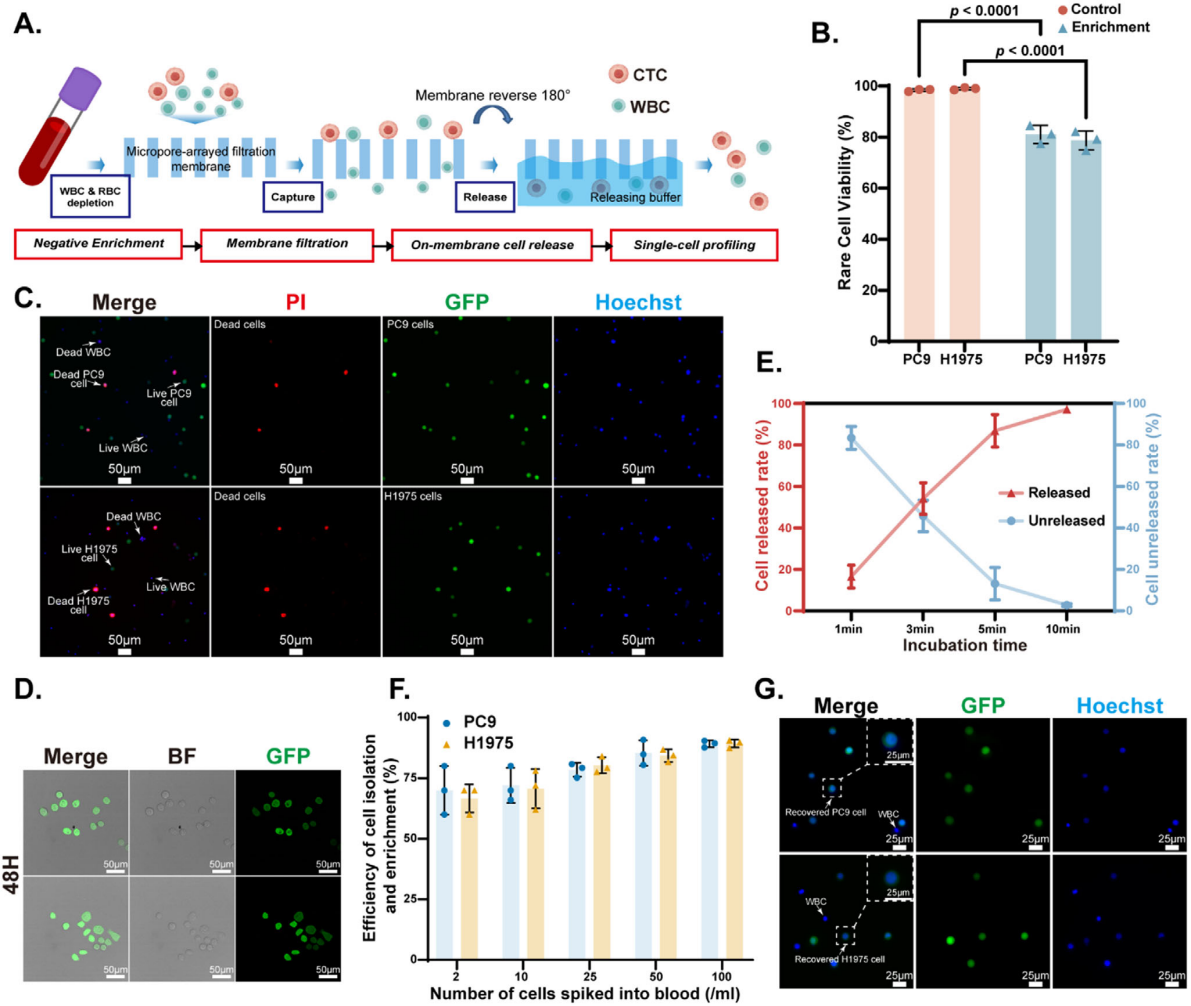
Performance evaluation of the self‐developed CTC sorting and capture platform. A) Schematic illustration of individual CTC enrichment using the self‐developed platform. B) Cell viability of PC9 and NCI‐H1975 cells enriched by the platform compared to control groups. Statistical analysis was performed using unpaired *t*‐tests (two‐tailed) for each cell line, with Šidák correction for multiple comparisons. C) Viability assessment of captured PC9 and NCI‐H1975 cells via propidium iodide (PI) staining (viable cells: green fluorescence; dead cells: red‐green overlay). D) Bright‐field and confocal fluorescence images of PC9 cells cultured in situ for 48 h post‐filtration, demonstrating platform compatibility with cell culture. E) Release efficiency of PC9 cells from a PVA‐coated micropore‐arrayed filtration membrane. F) Recovery rate of rare cells from 5 mL of peripheral blood using the platform. G) Fluorescence images of rare cells and leukocytes recovered from peripheral blood. B, E, F) Data presented as mean ± SD (*n =* 3).

As described in the Experimental section, we developed a CTC sorting and capture platform using an integrated approach that combines negative enrichment and physical separation techniques. To evaluate the platform's sorting performance, healthy human peripheral blood samples were spiked with PC9 and NCI‐H1975 lung cancer cell lines as rare cell models and processed through the platform for sorting and capture (CTC sorting and capture workflow shown in **Figure** **2**A). The viability of the captured PC9 and NCI‐H1975 cells was evaluated using live/dead cell staining assays. The results showed that the viability of captured PC9 and NCI‐H1975 cells was 81.1% ± 2.9% and 78.7% ± 3.0%, respectively (Figure 2B). Representative images of live/dead rare cells and background cells are shown in Figure 2C.

Previous studies have demonstrated that the edge‐to‐edge spacing of micropores is a critical factor affecting the viability of recovered cells.^[^ ^32^ ^]^ In this study, we used a Parylene C micropore‐arrayed filtration membrane with a pore size of 8 µm and pore spacing of 4 µm. The optical and scanning electron microscopy (SEM) images of this membrane are provided in Figure S1 (Supporting Information). Compared to other microporous membranes, the smaller pore spacing in our design enhances cell viability post‐capture, thereby significantly improving the platform's overall performance.

To further investigate the impact of the release process on cell viability, we evaluated the viability of cells after release from the platform. The results revealed that released cells maintained a viability of 87.2% ± 3.5% (Figure S2, Supporting Information), with no significant difference compared to the control group. This confirms that the release operation does not adversely affect cell viability. Additionally, to validate the long‐term viability of captured tumor cells, PC9 cells were cultured in situ for 48 h postcapture and release. As shown in Figure 2D, the cells exhibited strong adhesion and spreading morphology, indicating robust cell viability over an extended period. The release efficiency of the platform was also tested by capturing and releasing PC9 cells suspended in PBS. The results showed a release efficiency of 97.2% ± 0.6% (Figure 2E), confirming the platform's high capability for cell recovery.

To assess the platform's efficiency in recovering rare tumor cells from complex cellular backgrounds, fluorescently labeled PC9 or NCI‐H1975 cells were spiked into 5 mL of healthy human peripheral blood at concentrations of 2 to 100 cells mL^−1^. Postcapture cell counts revealed a recovery efficiency of 89.2% ± 1.3% when 100 rare cells per mL were spiked (Figure 2F). Representative images of captured fluorescently labeled cells are shown in Figure 2G.

Collectively, the platform enables efficient isolation of rare CTCs from peripheral blood while supporting their in vitro culture, thereby providing a basis for downstream analyses such as drug screening and genomic sequencing. Additionally, its capability for in situ culture of captured CTCs offers critical insights into their molecular and functional characteristics, advancing tumor metastasis research. A key innovation of this platform is the high‐efficiency, nondestructive release mechanism for captured CTCs,^[^ ^33^ ^]^ which achieved by a PVA coated micropore‐arrayed filtration membrane. This design allows gentle cell release at 37 °C while prevents issues such as deformation or physical entrapment. This platform outperforms existing CTC enrichment methods,^[^ ^34^, ^35^, ^36^, ^37^ ^]^ especially under low‐abundance conditions (≤100 cells mL^−1^ of blood). It enables rapid and sensitive enrichment of rare CTCs while maintaining high viability, fulfilling the essential requirements for liquid biopsy and multi‐omics analyses.

### 2.2 Isolate CTCs in Animal Models and Clinical Specimens

**Figure 3:**
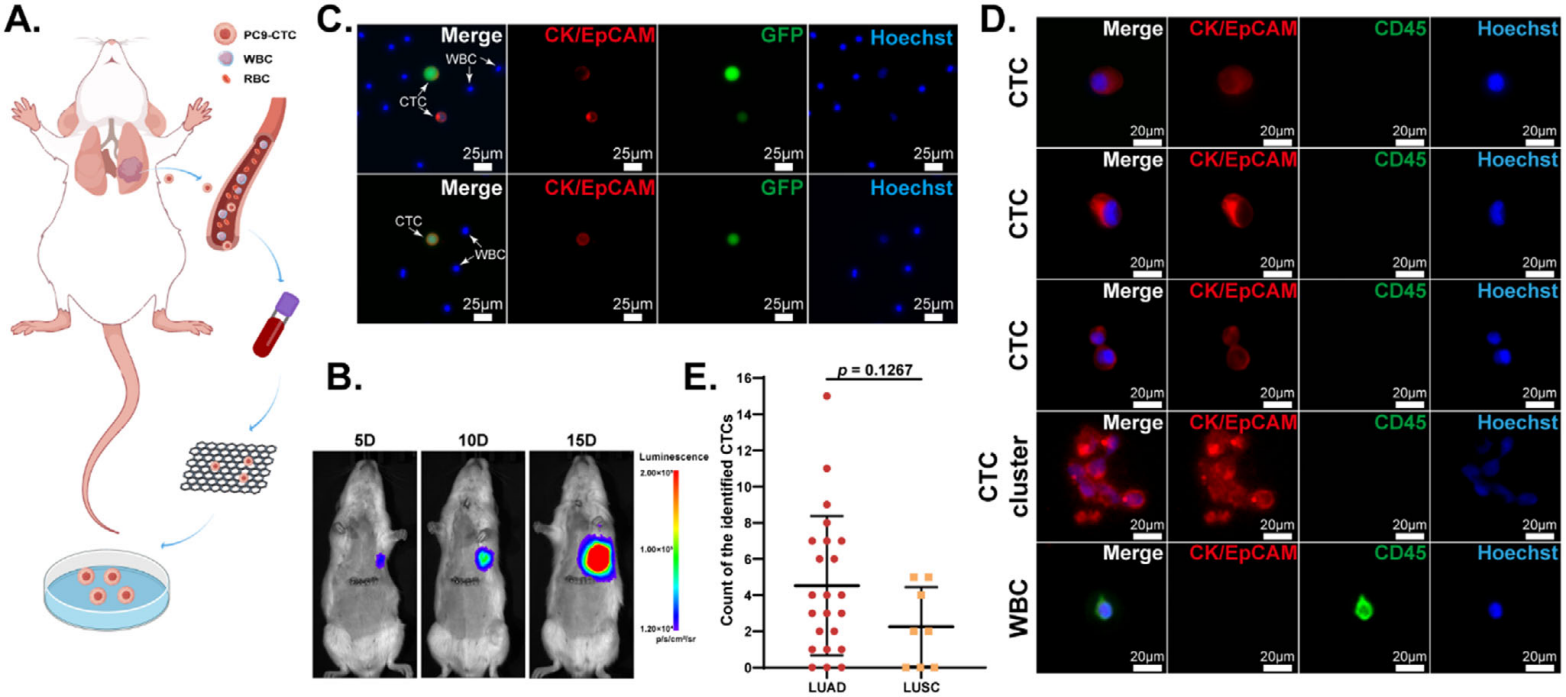
Isolate CTCs from animal models and clinical samples. A) Schematic of PC9‐CTC cell line derivation from orthotopic lung cancer rat models. B) Bioluminescence imaging of lung cancer rat models. C) Fluorescence images of CTCs and WBCs isolated from the peripheral blood of lung cancer rat models. D) Representative fluorescence images of CTCs (CK/EpCAM+) and WBCs (CD45+) captured from the peripheral blood of lung cancer patients. E) Distribution of CTC counts in 5 mL peripheral blood samples from 31 non‐small‐cell lung cancer patients. Statistical analysis: Data *E* is presented as mean ± SD (LUAD, *n* = 23; LUSC, *n* = 8). Significance was determined by two‐tailed Student's *t*‐test.

To validate the technical utility of our self‐developed CTC sorting and capture platform, we established an orthotopic lung cancer xenograft model for CTC isolation. As illustrated in **Figure** **3**A, luciferase‐expressing PC9 cells were orthotopically injected into the left lung parenchyma of highly immunodeficient SRG rats (which have functionally deleted *Rag2* and *Il2rg* genes on the Sprague‐Dawley background^[^ ^38^ ^]^) to generate primary tumors. Tumor progression was longitudinally monitored via IVIS Spectrum imaging on days 5, 10, and 15 after the injection (Figure 3B), and tumor size was measured to validate luminescence intensity (Figure S3, Supporting Information). After confirming lung tumor formation by imaging results, 1 mL of peripheral blood was collected via jugular vein puncture on day 16 for CTC isolation using self‐developed platform. Captured CTCs exhibited staining patterns of CK or EpCAM+/GFP+/Hoechst+, while remaining white blood cells (WBCs) displayed CK or EpCAM‐/GFP‐/Hoechst+ characteristics (Figure 3C). Through in vitro culture, we successfully established a CTC cell line (PC9‐CTC) with stable proliferative capacity. At the CTC collection timepoint, no extrapulmonary metastases were observed in the animal models, indicating that this CTC cell line represents circulating tumor cells shed from the primary lesion prior to metastatic dissemination.

Further functional assays were conducted to evaluate the metastatic potential of PC9‐CTC cells. Transwell assays demonstrated that PC9‐CTC cells exhibited higher migration and invasion capacity than parental PC9 cells (Figure S4A,B, Supporting Information). Additionally, 5‐ethynyl‐2′‐deoxyuridine (EdU) assays revealed faster proliferation rates in PC9‐CTC cells than PC9 cells (Figure S4C, Supporting Information). Further investigation of PC9‐CTC's epithelial‐mesenchymal transition (EMT) characteristics in the lung cancer organoid model uncovered a hybrid epithelial‐mesenchymal phenotype (Figure S5, Supporting Information). This suggests that EMT‐mediated acquisition of mesenchymal‐like properties contributes to the enhanced migratory and invasive capacities of PC9‐CTC cells.^[^ ^39^ ^]^

After validating the platform's efficacy in animal models, we conducted preliminary clinical evaluations using peripheral blood samples from 31 lung cancer patients (clinical information of the patients is provided in Table S1, Supporting Information). Captured cells were immunostained with anti‐CK, anti‐EpCAM, and anti‐CD45 antibodies. CTCs were identified as CK or EpCAM+/CD45‐/Hoechst+, while WBCs were identified as CK or EpCAM‐/CD45+/Hoechst+ (Figure 3D). No CTCs were detected in negative controls from five healthy volunteers and five patients with smoking history and benign pulmonary nodules (all pathologically confirmed after resection), confirming the assay's specificity (Figure S6, Supporting Information). CTC counts varied substantially among patients, with an average of 3.9 ± 3.5 CTCs per 5 mL of whole blood (Figure 3E). No significant correlation was observed between CTC counts and pathological subtypes. These results highlight the platform's high sensitivity for CTC detection in both animal models and clinical specimens, demonstrating its potential for practical liquid biopsy applications.

The PC9‐CTC cell line derived from lung cancer animal models exhibited enhanced invasive and migratory capacities compared to parental PC9 cells, highlighting CTCs’ role in metastasis.^[^ ^40^ ^]^ This supports the platform's utility for in vitro CTC cultivation and functional analysis, although optimized culture conditions are still needed for clinical CTC studies. While the exclusive use of female rats in this initial phase ensured model standardization, it may limit the generalizability of our findings. Given the known influence of biological sex on cancer research outcomes, we will incorporate male SRG rats in ongoing experiments to systematically evaluate sex‐specific effects on lung cancer model establishment, tumor progression, treatment response, and underlying mechanisms. In patient samples, CTC identification mainly relied on CK and EpCAM markers, which may overlook mesenchymal‐like CTCs and underestimate CTC heterogeneity.^[^ ^41^ ^]^ While our label‐free enrichment approach aims to capture diverse CTC phenotypes, epithelial‐biased identification criteria likely undercount actual CTC numbers. Future studies should develop broader CTC marker panels with improved specificity.

Our CTC sorting and capture platform demonstrates superior sensitivity in both animal models and clinical specimens, fulfilling clinical needs for noninvasive tumor diagnosis and therapeutic monitoring. Downstream molecular characterization of CTCs could elucidate associations between CTC phenotypic profiles and tumor aggressiveness, potentially revealing novel prognostic biomarkers and therapeutic molecular targets. Beyond rigorous analytical performance, this platform exhibits significant potential for clinical translation, promising to advance liquid biopsy technologies and improve cancer patient care.

### 2.3 Single‐Cell Metabolomic Profiling Uncovers Metastasis‐Specific Metabolic Heterogeneity in CTCs

To investigate the metabolic reprogramming of CTCs and its association with organ‐specific metastatic potential, we performed single‐cell metabolomic MS analysis on three cell populations: PC9‐CTC cells (isolated from orthotopic lung cancer rat models), Brm cells, and Bom cells (in prior studies,^[^ ^42^ ^]^ we established Brm and Bom lung cancer cell lines through intracardiac injection of PC9 cells into immunodeficient mice). These three types of cells represent circulating tumor cell populations shed from the primary site but not yet causing metastasis (PC9‐CTC), as well as CTC groups with high potential for Brm and Bom. Metabolic screening of three human lung adenocarcinoma cell lines with distinct metastatic tropisms identified potentially brain‐ or bone‐tropic metabolic signatures. A total of 181 single cells (58 PC9‐CTC, 60 Bom, and 63 Brm cells) were analyzed using the nanoESI‐APCI‐HRMS platform, resulting in the identification of 390 unique metabolites (Table S2, Supporting Information).

**Figure 4:**
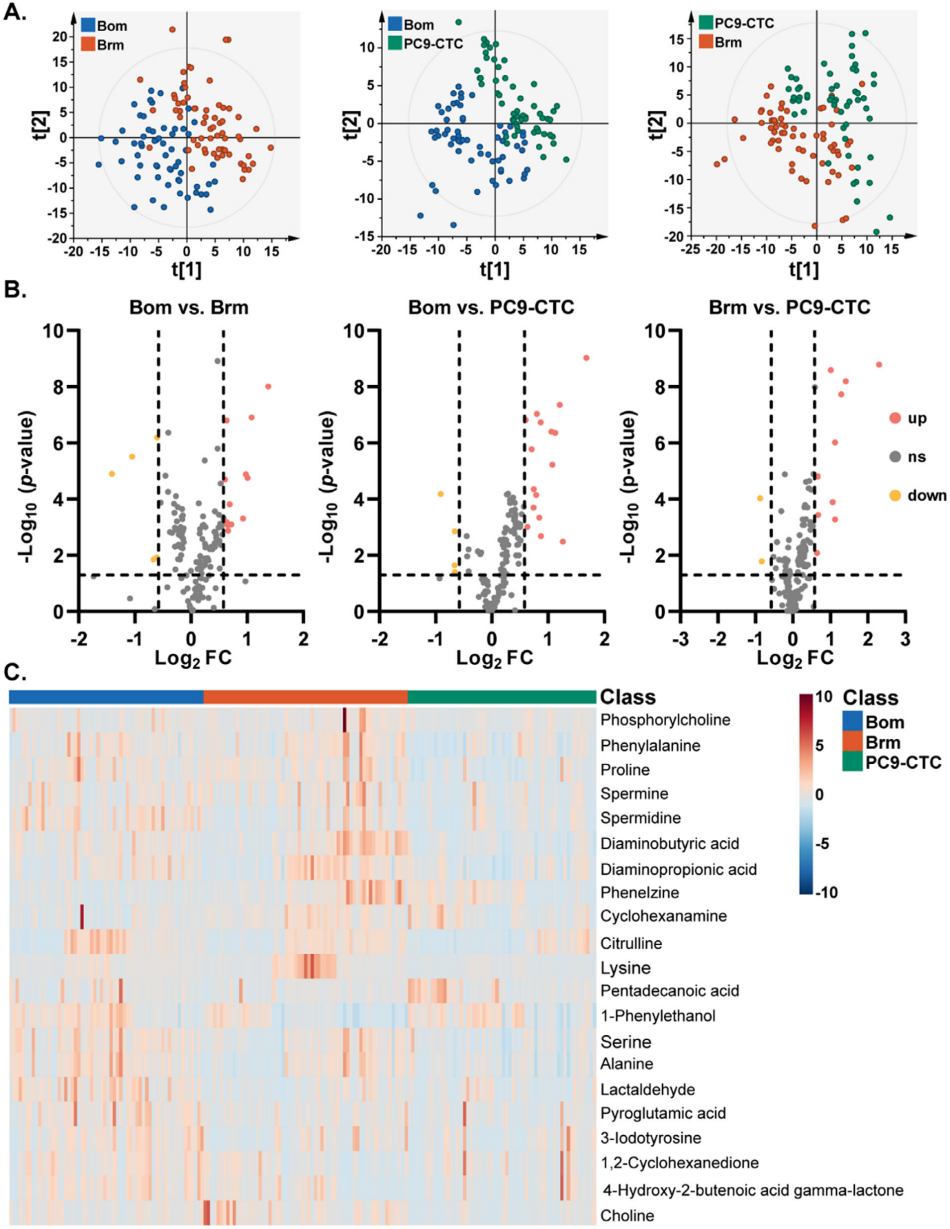
Metabolic profiles of CTCs and their association with metastatic potential. A) PLS‐DA plots of Bom/Brm, Bom/PC9‐CTC, and Brm/PC9‐CTC cell lines (Bom/Brm: R^2^X (cum) = 0.534, R^2^Y (cum) = 0.890, Q^2^ (cum) = 0.671; Bom/PC9‐CTC: R^2^X (cum) = 0.598, R^2^Y (cum) = 0.858, Q^2^ (cum) = 0.584; Brm/PC9‐CTC: R^2^X (cum) = 0.739, R^2^Y (cum) = 0.875, Q^2^ (cum) = 0.748). B) Volcano plots of selected metabolites (VIP ≥ 1) in Bom versus Brm, Bom versus PC9‐CTC, and Brm versus PC9‐CTC cell lines. Red and blue dots represent increased and decreased metabolites, respectively (FC ≥ 1.5, *p* < 0.05; significance determined by Mann‐Whitney *U* test). **C)** Heatmap of the relative intensities of differential metabolites in Bom, Brm, and PC9‐CTC cell lines.

Pairwise partial least squares discriminant analysis (PLS‐DA) revealed distinct clustering patterns among Bom, Brm, and PC9‐CTC cells (**Figure** **4**A), with permutation tests confirming model robustness (Figure S7, Supporting Information). Based on the variable importance in projection (VIP) values derived from the PLS‐DA models, an initial set of differential metabolites (VIP ≥ 1) was defined, followed by rigorous filtering via volcano plot analysis with criteria of fold change (FC) ≥ 1.5 and *p* ≤ 0.05 (Figure 4B). Kyoto Encyclopedia of Genes and Genomes (KEGG) pathway enrichment analysis was performed to identify the metabolic pathways associated with the differential metabolites (Figure S8, Supporting Information). The analysis revealed significant differences in several key metabolic pathways among the cell types. For instance, the arginine and proline metabolic pathway was more prominent in Bom cells compared to Brm cells, indicating its potential role in bone metastasis.^[^ ^43^ ^]^ Differential metabolites were also observed in tyrosine metabolism between Bom and Brm cells, suggesting distinct metabolic adaptations in these metastatic variants. In the comparison between Bom and PC9‐CTC cells, the arginine biosynthesis pathway was found to be more prominent,^[^ ^44^ ^]^ and significant differences were observed in glycine, serine, and threonine metabolism.^[^ ^45^ ^]^ In the comparison between Brm and PC9‐CTC cells, phenylalanine, tyrosine, and tryptophan biosynthesis was more pronounced in Brm cells, revealing potential metabolic signatures associated with brain metastasis. A heatmap visualizing the differential metabolites corresponding to the identified KEGG pathways (Figure 4C) clearly illustrated the distinct metabolic profiles of Bom, Brm, and PC9‐CTC cells, highlighting the metabolic heterogeneity associated with different metastatic potentials.

The distinct metabolic profiles observed in CTCs and metastatic variants provide valuable insights into the metabolic adaptations that drive metastasis. Our results suggest that targeting specific metabolic pathways could inhibit metastatic progression by disrupting the adaptive capabilities of tumor cells. Future studies should focus on validating these metabolic signatures in larger cohorts and exploring their potential as diagnostic biomarkers or therapeutic targets.

### 2.4 Metabolite‐Driven Classification Model Identifies CTC Subgroups with Distinct Metastatic Risks

To establish a clinically practicable framework for stratifying metastatic risk, we developed a metabolite‐based classification model utilizing the metabolic heterogeneity of CTCs. As described previously, metabolic reprogramming in CTCs reflect their adaptive capabilities and organotropism during metastasis.^[^ ^46^ ^]^ We hypothesized that intrinsic metabolic differences among CTC subpopulations could serve as discriminative markers to categorize CTCs into functionally distinct subgroups.

To assess the clinical relevance of the identified metabolites, we collected blood samples from 30 patients with lung adenocarcinoma, including 10 with brain metastasis, 10 with bone metastasis, and 10 with nonmetastatic lung cancer. Using our CTC sorting and capture platform, a total of 120 CTCs was isolated from these patients. The primary clinical characteristics and biomarker levels of the patients are summarized in Tables S3 and S4 (Supporting Information). In the training cohort, the number of CTCs detected per 5 mL of blood ranged from 2 to 11, with a frequency distribution of 2 CTCs (10%), 3 CTCs (33.3%), 4 CTCs (30.0%), and ≥5 CTCs (26.7%). Notably, there was no significant difference in total CTC counts between the nonmetastasis group (median: 3; range: 2–5) and the metastasis group (median: 4; range: 2–11) (*p* = 0.1170).

**Figure 5:**
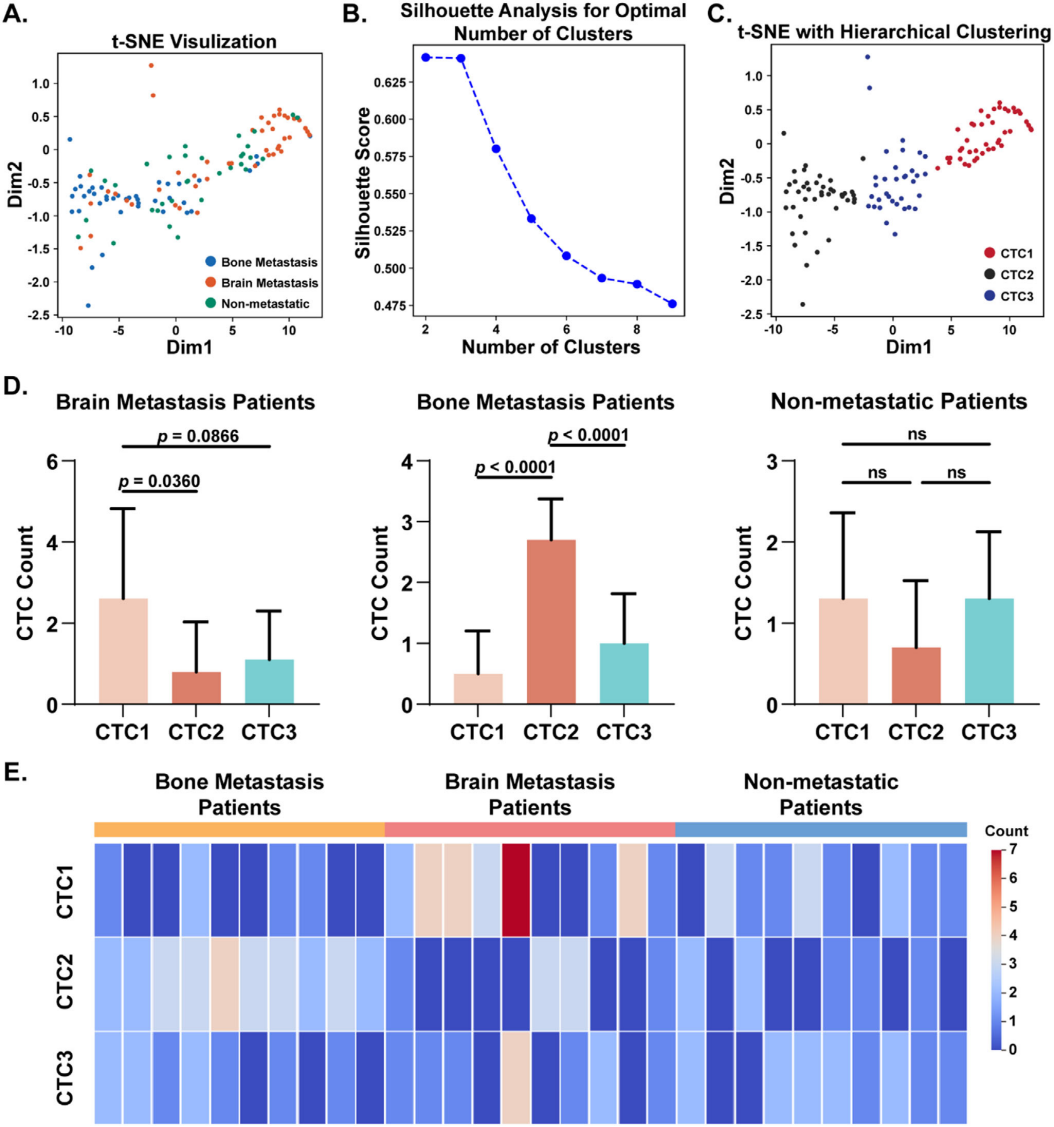
The heterogeneity of CTCs and their potential utility in identifying metastatic risk in lung cancer patients. A) t‐SNE visualization of CTCs from patients. B) Silhouette analysis based on dimension reduction using t‐SNE. C) t‐SNE visualization of CTCs labeled by hierarchical clustering. D) Distributions of CTC subgroups in patients. E) Heatmap of CTC subgroup counts in patients. Statistical analysis: Data in *D* is presented as mean ± SD (*n* = 10). Significance was determined by one‐way Analysis of Variance.

The isolated CTCs were aliquoted into 200 µL Eppendorf tubes containing 1 µL of 150 mM ammonium formate solution and stored at −80 °C. Prior to MS analysis, samples were lyophilized under nitrogen flow, reconstituted in 1 µL of methanol containing deuterated phenylalanine (d5‐Phe, 1 µg mL^−1^) as an internal standard and subjected to nanoESI‐APCI‐HRMS. The obtained raw MS data were normalized to internal standard intensity and aligned with reference m/z values of the 390 previously identified metabolites. To resolve CTC heterogeneity, we applied t‐distributed stochastic neighbor embedding (t‐SNE), a nonlinear dimensionality reduction algorithm, to visualize metabolic similarities among CTCs. As shown in **Figure** **5**A, CTCs from different patient cohorts exhibited partial overlap but formed three tentative clusters, reflecting both interpatient variability and intrinsic metabolic divergence.

To objectively define CTC subgroups, we performed silhouette analysis on t‐SNE embeddings, which identified three optimal clusters (Figure 5B). Hierarchical clustering confirmed this partitioning, categorizing CTCs into three subgroups: CTC1 (*n =* 44), CTC2 (*n =* 42), and CTC3 (*n =* 34) (Figure 5C; Table S5, Supporting Information). Subgroup distributions across patients revealed striking clinical correlations: CTC1 was predominantly enriched in brain metastasis patients, CTC2 was notably prevalent in bone metastasis patients, and CTC3 showed no significant association with metastatic status (Figure 5D; Table S6, Supporting Information). These findings indicate that higher CTC1 and CTC2 counts are associated with brain and bone metastasis, respectively. In non‐metastatic patients, increased CTC1 levels may suggest a potential risk of brain metastasis, while elevated CTC2 levels may indicate a possible risk of bone metastasis. Conversely, predominant CTC3 counts are likely to imply a lower short‐term metastatic risk. Figure 5E displays the distribution of each CTC subgroup within individual patients, indicating that the metastasis‐associated CTC1 and CTC2 subgroups are indeed more broadly distributed in the corresponding metastatic populations. To validate these observations, Uniform Manifold Approximation and Projection (UMAP) was applied as an alternative dimensionality reduction method. UMAP revealed a CTC distribution pattern similar to that observed with t‐SNE. Subsequent silhouette analysis and hierarchical clustering of UMAP embeddings further confirmed that CTC subgroup distributions closely matched those derived from t‐SNE (Figure S9, Supporting Information). These complementary analyses collectively reinforce the robustness and interpretation of our findings. The presence of CTC1 or CTC2 subgroups in patients with primary tumors could serve as an early warning of tumor metastasis, enabling timely adjustments to therapeutic strategies.

These outcomes underscore the potential of CTC subgroup analysis as a valuable tool for predicting metastatic risk and guiding personalized treatment strategies in patients with lung cancer.

### 2.5 CTC Subgroup Abundance Predicts Organ‐Specific Metastasis Risk in Lung Cancer

**Figure 6:**
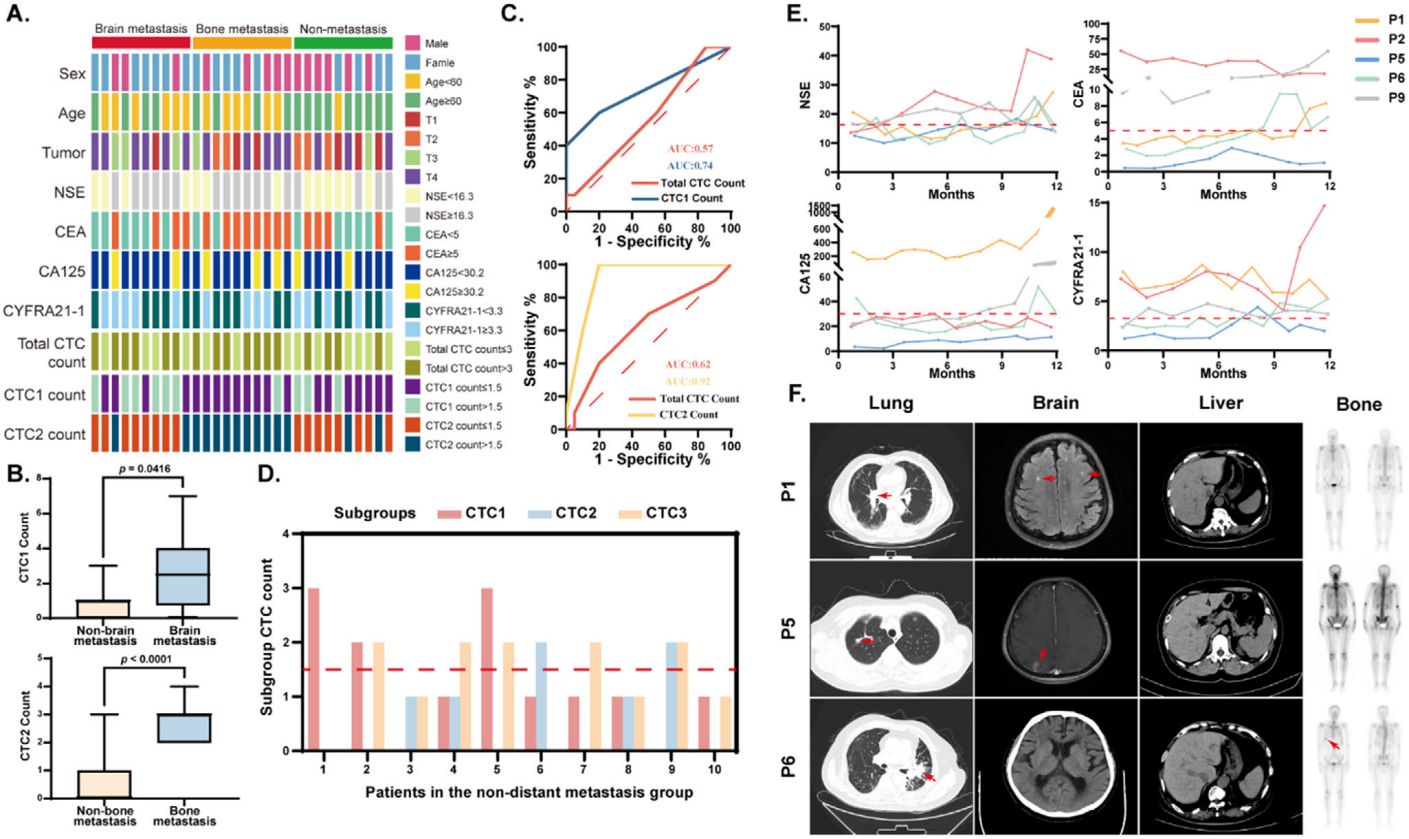
Predictive potential of metastasis‐associated CTC subgroups for distant lung cancer metastasis. A) Correlation between patient metastasis status (distant metastasis vs non‐distant metastasis), clinical characteristics, biomarker levels (NSE, CEA, CA‐125, and CYFRA21‐1), and CTC subgroup counts (CTC1, CTC2, total CTCs) in the test cohort. B) Comparison of CTC1 and total CTC counts between brain metastasis‐positive and ‐negative groups, and CTC2/total CTC counts between bone metastasis‐positive and ‐negative groups in the training cohort. Box plots show medians (horizontal lines), 25th–75th percentiles (boxes), and ranges (whiskers). Statistical significance was determined by two‐tailed Student's *t*‐test. C) ROC analysis of CTC1, CTC2, and total CTC counts for predicting brain or bone metastasis in the training cohort. D) Distribution of CTC1, CTC2, and CTC3 counts in non‐distant metastasis patients. E) Changes in tumor biomarkers (NSE, CEA, CA‐125, and CYFRA21‐1) during follow‐up for five patients with a high risk of distant metastasis (P1, P2, P5, P6, and P9). The cut‐off values for each biomarker are indicated by red lines. F) Representative CT and ECT images of the patients (P1, P5, and P6) showing metastatic lesions (red arrows) detected during follow‐up.

Emerging evidence indicates that CTCs can be detected even in the early stages of lung cancer, and their clustering forms often possess a higher metastatic capacity compared to individual CTCs. Moreover, CTCs exhibit remarkable adaptability through diverse phenotypes, enabling them to facilitate metastasis and develop resistance to therapies. However, the specific subgroups of CTCs and their correlation with different metastatic risks in lung cancer have not been fully explored. This study aimed to investigate the association between CTC subgroups and lung cancer metastasis risk, as well as their correlations with clinical characteristics and conventional tumor biomarkers. The results demonstrated no significant associate between metastasis risk and factors such as sex, age, primary lung cancer status, or biomarkers including NSE, CEA, CA‐125, and CYFRA21‐1 (**Figure** **6**A). However, elevated CTC1 subgroup counts (*p* = 0.0416) and CTC2 subgroup counts (*p* < 0.0001) were strongly linked to the risks of brain and bone metastasis, respectively (Figure 6B). Receiver Operating Characteristic (ROC) curve analysis determined the optimal cutoff thresholds for CTC1 and CTC2 subgroups as 1.5 based on Youden's index (Figure 6C). Patients with CTC1 or CTC2 counts exceeding these thresholds exhibited a poorer prognosis and higher metastasis risk (Figure 6A). In terms of predictive performance, the area under the ROC curve (AUC) for CTC1 in predicting brain metastasis and CTC2 in predicting bone metastasis were 0.74 and 0.92, respectively. These values significantly outperformed the predictive performance of total CTC counts (Figure 6C). Univariate and multivariate logistic regression analyses further confirmed that CTC subgroup counts are independent predictors of lung cancer metastasis (Tables S7 and S8, Supporting Information).

Additionally, a prospective study was conducted on 10 lung cancer patients without distant metastasis in the cohort. Basic clinical information is summarized in Table S3 (Supporting Information). The median CTC count was 3/5 mL of blood (range: 2–5). Subgroup classification based on CTC metabolic phenotypes revealed that cases 1, 2, and 5 had CTC1 counts above the threshold (indicating elevated brain metastasis risk), while cases 6 and 9 had elevated CTC2 counts (indicating increased bone metastasis risk) (Figure 6D). During the 1‐year follow‐up, cases 1 and 5 developed brain metastasis at 9 and 11 months, respectively, and case 6 developed bone metastasis at 6 months. The remaining 7 patients showed no signs of extrapulmonary metastasis (Figure 6F), validating the feasibility of metabolic‐based CTC subgroup classification for metastasis prediction. In contrast, conventional tumor biomarkers showed no significant prognostic correlations (Figure 6E).

In clinical practice, CTCs, as a critical component of “liquid biopsy”, have demonstrated significant potential in the diagnosing, prognosing, and monitoring solid tumors.^[^ ^47^ ^]^ However, most studies focus only on the correlation between CTC counts and prognosis, ignoring the substantial heterogeneity of CTCs across patients and disease stages,^[^ ^48^ ^]^ which limits their broader clinical utility. Single‐cell sequencing technologies offer unprecedented opportunities to comprehensively dissect CTC heterogeneity, elucidate molecular mechanisms of metastasis, and identify novel therapeutic strategies.^[^ ^12^, ^49^ ^]^

Metabolic reprogramming is a key driver of tumor progression and a key contributor to intra‐ and intercellular heterogeneity.^[^ ^50^ ^]^ Thus, single‐cell metabolomic profiling of CTCs may uncover the link between their heterogeneity and metastatic propensity.^[^ ^51^ ^]^ This study employed single‐cell metabolomics to classify CTCs into distinct subgroups based on characteristic metabolites, revealing that CTC1 and CTC2 subgroup abundances closely correlated with patient prognosis. In prospective validation, metabolic phenotype‐based CTC classification outperformed total CTC counts and conventional biomarkers in predicting metastasis within 1 year, suggesting single‐cell metabolomics as a powerful complement to CTC enumeration for enriched prognostic insights. However, the molecular mechanisms driving these metabolic alterations remain unclear and require further investigation.

While our study has demonstrated the potential of single‐cell metabolomics to uncover metastasis‐specific metabolic heterogeneity in CTCs and to stratify CTCs into subgroups with distinct metastatic risks, it is important to acknowledge several limitations in this study. First, the deeper metabolic regulatory mechanisms underlying brain‐ and bone‐tropic metastasis in lung cancer CTCs remain to be fully elucidated. Further mechanistic studies are warranted to explore the metabolic drivers of organotropic metastasis and their clinical implications. Second, the relatively small sample size of our study cohort may limit the statistical power and generalizability of our findings. Third, while we specifically enrolled patients with brain or bone metastases, some cases also presented with concurrent lymph node involvement, which warrants further investigation regarding its potential influence on distant metastasis prediction. Additionally, the absence of longitudinal data limits our ability to assess the dynamic changes in CTC metabolites during disease progression and treatment. Incorporating longitudinal sampling will be essential to elucidate temporal dynamics of CTC metabolism and its relationship with metastasis.

Future studies will focus on validating and expanding the clinical utility of our CTC metabolite‐based classification system. One promising direction is to apply this analysis to a prospective cohort of locally advanced lung cancer patients. This will allow us to evaluate whether the identified metabolomic signatures can predict site‐specific metastasis before it occurs. Such studies could provide valuable insights into the early metastatic events and help identify high‐risk patients for specific metastasis, enabling timely interventions and personalized treatment strategies. Additionally, we aim to explore the dynamic changes in CTC metabolites during disease progression and treatment, which may further enhance our understanding of the metabolic mechanisms underlying metastasis and improve the predictive accuracy of our model. Another important area for future research is the integration of multi‐omics approaches with single‐cell metabolomics. Combining metabolomic data with genomic, transcriptomic, and proteomic profiles could provide a more comprehensive understanding of the biological mechanisms driving metastasis.^[^ ^52^, ^53^, ^54^ ^]^ This integrative approach may identify novel therapeutic targets and biomarkers that can be translated into clinical practice.

Taken together, this study established a metabolic phenotype‐based CTC classification system by integrating CTC enrichment, single‐cell metabolomics, and metastasis‐specific metabolic profiles. This marks a critical step toward precise prediction of lung cancer metastasis risk. Although a larger cohort validation and functional characterization of key metabolites are needed, this approach offers a novel perspective for integrating metabolomics into clinical decision‐making. With advancing technologies, single‐cell molecular profiling is set to assume a growing role in oncology. Elucidating the interplay between CTC heterogeneity and disease progression will drive liquid biopsy from quantitative counting to qualitative analysis, advancing precision medicine. Furthermore, methodologies to identify functional CTC subgroups at the single‐cell level may open new avenues for cancer biology, facilitating the discovery of prognostic markers and therapeutic targets.

## 3 Conclusion

This study introduces a novel approach to classify CTCs based on their metabolic profiles, offering a more precise prediction of lung cancer metastasis. By integrating a self‐developed CTC sorting and capture platform with single‐cell metabolic MS analysis, we have successfully established a classification system that categorizes CTCs into distinct subgroups associated with metastatic potential. This innovative method not only demonstrates superior predictive power compared to traditional clinical indicators and total CTC counts but also highlights the potential of single‐cell metabolic profiling in uncovering new therapeutic targets and prognostic markers.

The key findings and contributions of this study are as follows: i) Development of a platform for high‐viability capture and effective release of rare CTCs from peripheral blood, which supports downstream analyses; ii) Identification of differential metabolites linked to metastatic potential through single‐cell metabolic profiling; iii) Establishment of a model that categorizes CTCs into subgroups with distinct metastatic risks, validated using clinical specimens; iv) Prospective follow‐up confirmed the accuracy of this metabolite‐based classification system in predicting metastasis risk.

In summary, this work represents a significant advancement in liquid biopsy and precision medicine, offering a more personalized approach to managing lung cancer. Future research will focus on validating this approach in larger cohorts and exploring the underlying molecular mechanisms. This study paves the way for integrating single‐cell metabolomics into clinical decision‐making to enhance patient care.

## 4 Experimental Section

The detailed procedures of certain experiments are provided in the Supporting Experimental Section.

### Materials and Reagents

A 0.25% Trypsin‐EDTA, Antibiotic‐Antimycotic (100X), Dulbecco's Modified Eagle's Medium (DMEM), Fetal Bovine Serum (FBS) and phosphate buffered saline (PBS) were purchased from Meilunbio (Dalian, China). Ammonium formate was purchased from Sigma Aldrich (St. Louis, MO). Ultrapure water was produced by Milli‐Q water purification system (Millipore, Bedford, MA).

### Clinical Characteristics of Patients with Lung Cancer

This study was conducted at the Second Affiliated Hospital of Dalian Medical University from March 2023 to May 2024, with approval from the Institutional Ethics Committee (Approval No. 128, 2023; Second Affiliated Hospital of Dalian Medical University). All participants provided written informed consent. A total of 61 non‐small cell lung cancer (NSCLC) patients were enrolled, of whom 31 were used for clinical validation of the CTC sorting and capture platform, and 30 were for constructing a single‐cell metabolic profiling model of lung cancer CTCs. Inclusion criteria for patients in the metabolic profiling cohort were as follows: 1) Age ≥ 18 years; 2) Pathologically confirmed lung adenocarcinoma; 3) Imaging‐confirmed brain or bone metastasis; 4. No history of other malignancies or major comorbidities within the past five years. After excluding cases with CTC counts < 2, patients were stratified into three groups based on metastatic status: brain metastasis group (*n* = 10), bone metastasis group (*n* = 10), and nonmetastatic group (*n* = 10). Patients in the non‐metastatic group underwent prospective follow‐up for one year, with quarterly assessments by specialists to document metastasis occurrence and location. Clinical characteristics collected included age, sex, TNM stage, and serum biomarkers (NSE, CEA, CA‐125, CYFRA21‐1).

### Cells and Cell Culture

Human lung adenocarcinoma cell lines PC9 (RRID: CVCL_B260) and NCI‐H1975 (RRID: CVCL_1511) were purchased from the Chinese Academy of Medical Sciences (Beijing, China). Brain metastasis‐derived sublines (Brm) and bone metastasis‐derived sublines (Bom) were previously established by the research group through experimental protocols.^[^ ^42^ ^]^ Parental PC9, NCI‐H1975, Brm, Bom, and circulating tumor cell sublines (PC9‐CTC) were cultured in RPMI 1640 medium (Meilunbio, Dalian, China) supplemented with 10% FBS and 1% penicillin/streptomycin (P/S). All cell lines were maintained at 37 °C in a humidified incubator with 5% CO₂. All cell lines tested negative for Mycoplasma contamination and were authenticated by short tandem repeat (STR) analysis upon acquisition. Prior to cryopreservation, cells received Mycoplasma prophylactic treatment; following resuscitation, they were maintained in prophylaxis‐free culture medium for 5–7 days to confirm the absence of prophylactic effects on experimental outcomes.

### CTC Sorting and Capture

For CTC sorting, fresh whole blood samples were treated with RosetteSep Human CD45 Depletion Cocktail (STEMCELL Technologies, USA). The samples were mixed thoroughly and incubated at room temperature for 20 min to promote specific crosslinking between leukocytes and erythrocytes. The treated blood samples were then diluted 1:1 with buffer (Hank's Balanced Salt Solution containing 2% FBS). The diluted blood was carefully layered onto a pre‐prepared density gradient medium and centrifuged at 1200 g for 20 min. After centrifugation, the cell layer at the interface between the density gradient medium and diluted plasma was gently aspirated using a sterile pipette. For CTC capture, a micropore‐arrayed filtration membrane was secured in a custom‐designed holder and pretreated with 75% ethanol, followed by thorough rinsing with Hank's Balanced Salt Solution. The enriched cell suspension was transferred onto the membrane surface using a pipette. After complete filtration, CTCs and residual blood cells were retained on the membrane. Captured cells were gently washed with buffer to remove nonspecifically adhered blood cells. Finally, the membrane was removed from the holder, inverted, and immersed in buffer at 37 °C for 10 min. As the PVA coating dissolved, the captured cells were released into the buffer. All procedures were performed in a laminar flow hood to maintain sterility. Sample processing was completed within 2 h to maximize cell viability. Processed samples were either immediately analyzed or temporarily stored at 4 °C.

### Sorting and Capture platform Performance Toward Rare Tumor Cells

To evaluate the platform's ability to maintain the viability of rare tumor cells during peripheral blood processing, healthy donor blood samples were spiked with GFP‐labeled PC9 and NCI‐H1975 cells (100 cells mL^−1^) and processed through the CTC sorting and capture platform. Captured cells were then subjected to in situ staining using a commercial live/dead cell viability kit (Solarbio Science & Technology, Beijing, China), with propidium iodide (PI, dead cell marker, red fluorescence). Rare cells displaying red fluorescence were observed and counted under a fluorescence microscope to calculate short‐term survival rates. A control group (untreated spiked cells) and the experimental group (processed cells) were maintained under identical conditions for comparison. To assess long‐term viability, captured PC9 cells were cultured in situ for 48 h, followed by evaluation of their morphology, adhesion, and spreading behavior.

To validate the recovery efficiency of the CTC sorting and capture platform for target cells in complex cellular backgrounds, healthy donor whole blood samples (5 mL) were spiked with varying numbers (10, 50, 125, 250, and 500) of PC9 and NCI‐H1975 cells to test recovery rates at concentrations of 2, 10, 25, 50, and 100 mL. For cell counts exceeding 100, spiking was performed via serial dilution, while 50‐cell aliquots were precisely added using a microscope‐guided micropipette and micromanipulator (Eppendorf, Hamburg, Germany). To facilitate fluorescence microscopy‐based observation and recovery rate quantification, PC9 and NCI‐H1975 cells were pre‐labeled with fluorescent markers. During spiking, tumor cells were resuspended in RPMI 1640 medium supplemented with 10% FBS to maintain viability during the precise, time‐sensitive pipetting process.

### Construction of Lung Cancer Model

A lung cancer animal model was established using female SRG rats (6–8 weeks old). PC9‐GFP‐Luc cells (expressing green fluorescent protein and luciferase) were employed to monitor tumor growth via bioluminescence imaging. A cell suspension containing 5 × 10^6^ cells/rat in 50 µL was mixed 1:1 with Matrigel matrix (Corning, New York, USA) and injected into the lungs of immunodeficient rats via orthotopic implantation. In vivo analysis of lung colonization was performed using bioluminescence imaging technology, with images acquired every 5 days starting 5 days postinjection via the IVIS imaging system. Peripheral blood was collected via the jugular vein prior to euthanasia. All animal experiments were approved by the Dalian Medical University Animal Ethics Committee (AEE23117). Following peripheral blood collection, all experimental animals were euthanized under anesthesia.

### Detection of CTCs in Blood Samples

To validate the effectiveness of the CTC sorting and capture platform in practical applications and enable downstream CTC analysis, peripheral blood samples from lung cancer animal models and non‐small cell lung cancer (NSCLC) patients was processed. To confirm the specificity of the capture and staining protocols, blood samples were collected from healthy volunteers (*n* = 5) and patients (*n* = 5) with a smoking history and pathologically confirmed benign pulmonary nodules (post‐resection) as negative controls. Rat (2 mL) and human (5 mL) peripheral blood samples were drawn into EDTA‐containing vacuum blood collection tubes. For patient samples, unfixed CTCs were labeled with Alexa Fluor 594‐conjugated pan‐cytokeratin (CK, 1:100), Alexa Fluor 594‐conjugated anti‐human CD326 (EpCAM, 1:100), Alexa Fluor 488‐conjugated CD45 (1:100), and Hoechst 33 342 (1:100). Cells were observed and counted using fluorescence microscopy, with CTCs defined as CK+/EpCAM+, CD45−, and Hoechst+. For subsequent single‐cell metabolomic analysis, individual CTCs from patients were isolated under direct microscopic visualization using a micropipette and micromanipulator (Eppendorf, Hamburg, Germany) and transferred to sample preservation tubes. All procedures were completed within 2 h to ensure cell viability.

### Mass Spectrometric Single‐Cell Metabolomics Analysis

The nanoelectrospray ionization‐atmospheric pressure chemical ionization (nanoESI‐APCI) analysis procedure was conducted as described in previous publication.^[^ ^55^ ^]^ Briefly, cells were cultured in 6 cm dishes until they reached 80% confluence. The culture medium was then removed, and the cells were washed twice with 4 mL PBS to remove residual medium, followed by the addition of 5 mL PBS. Afterward, the dish was transferred to the stage of a CKX53 microscope (Olympus, Tokyo, Japan), where a nanoESI micropipette preloaded with 10 µL of 150 mm ammonium formate solution was mounted on a 3‐D micromanipulator (MP‐225, Sutter Instrument, Novato, CA). The micropipette was obtained by pulling a borosilicate glass capillary tube (I.D. 0.86 mm, O.D. 1.5 mm) to form a tip with 1 µm via a P‐1000 puller (Sutter Instrument, Novato, CA). Under microscopic guidance, the tip was carefully moved toward the cells. Once the tip contacted the cell surface, an air pump was activated to create a negative pressure at the tail of the micropipette for the extraction of cytoplasm. The extraction was maintained for 90 s. After completion, the micropipette was unloaded and transferred to the homemade nanoESI‐APCI platform, where the APCI source and pulsed high‐voltage were activated to perform nanoESI‐APCI analysis on a QE‐HF mass spectrometer. The MS parameters were set as follows: the capillary temperature was set to 275 °C, with both gas flow and voltage set to 0. The RF‐lens was adjusted to 50. Data acquisition was performed in full‐scan mode with a mass range of 70–1050 m z^−1^.

### Data Processing and Statistical Analysis

Custom *R* or Python programs were developed to process raw single‐cell metabolomics data, including peak alignment, stable ion feature selection, and metabolite identification. Detailed information is provided in the Supporting Information.

## Conflict of Interest

The authors declare no conflict of interest.

## Author Contributions

Y.X., X.H., and Y.Y. contributed equally to this work. Three authors were involved in experimental design, experimental execution, data collection, statistical analyses, and manuscript writing. J.W., W.L., and C.Y. collected all biological samples. X.S. critically revised the manuscript for important intellectual content and approved the version to be published. W.Q. and L.W. assisted with data interpretation. M.L. and Y.J. assisted with the CTC sorting and capture. Q.W., G.X., C.H. and W.W. are the guarantors of this work and, as such, had full access to all the data in the study and take responsibility for the integrity of the data and the accuracy of the data analysis.

## Supporting information



Supporting Information

## Data Availability

The data that support the findings of this study are available in the supplementary material of this article.
