# Supplementary material for: Prediction of Lung Cancer Metastasis Risk Based on Single‐Cell Metabolic Profiling of Circulating Tumor Cells
Source: Adv Sci (Weinh). 2025 Jul 18;12(39):e08878. doi: 10.1002/advs.202508878 (PMC12533299; doi:10.1002/advs.202508878)
Supplement: Supplementary file 1 — Supporting Information [file ADVS-12-e08878-s001.docx]

**Supporting Information**

**Single-Cell Metabolic Profiling of Circulating Tumor Cells for Predicting Lung Cancer Metastasis Based on Metabolic Heterogeneity**

*Yang Xu^1,3‡^, Xuesen Hu^2,4,5‡^, Yuan yuan^1,3‡^, Wenwen Liu^1,3^, Jian Wang^3,6^, Chunhui Yang^1,3^, Xianzhe Shi^2,4,5^, Wangshu Qin^2,4,5^, Liliang Wen^2,4,5^, Manqing Lin^1,3^, Yinuo Jin^1,3^, Wei wang^7,8*^, Chunxiu Hu^2,4,5*^, Guowang Xu^2,4,5*^, Qi Wang^1,3*^*

^1^ Department of Respiratory Medicine, the Second Affiliated Hospital of Dalian Medical University, Dalian 116023, China

^2^ State Key Laboratory of Medical Proteomics, Dalian Institute of Chemical Physics, Chinese Academy of Sciences, Dalian 116023, China

^3^ Cancer Translational Medicine Research Center, the Second Affiliated Hospital of Dalian Medical University, Dalian 116023, China

^4^ University of Chinese Academy of Sciences, Beijing 100049, China

^5^ Liaoning Province Key Laboratory of Metabolomics, Dalian 116023, China

^6^ Medical College of Tarim University, Aral 843300, China

^7^ School of Integrated Circuits, Peking University, Beijing 100871, China

^‡^ These authors contribute equally to this work.

^*^ Correspondence to:

Prof. Dr. Wei Wang, School of Integrated Circuits, Peking University, Beijing 100871, China. E-mail: w.wang@pku.edu.cn

Prof. Dr. Chunxiu Hu, State Key Laboratory of Medical Proteomics, Dalian Institute of Chemical Physics, Chinese Academy of Sciences, Dalian 116023, China. Tel.: 0086-411-84379532, Fax: 0086-411-84379559. E-mail: hucx@dicp.ac.cn

Prof. Dr. Guowang Xu, State Key Laboratory of Medical Proteomics, Dalian Institute of Chemical Physics, Chinese Academy of Sciences, Dalian 116023, China. Tel./Fax: 0086-411-84379530. E-mail: xugw@dicp.ac.cn

Prof. Dr. Qi Wang, Department of Respiratory Medicine, the Second Affiliated Hospital of Dalian Medical University, Dalian 116023, China. E-mail: wqdlmu@163.com

Table of Contents

Experimental section S4

**Supporting Figures:**

Figure S1. The optical and scanning electron microscopy (SEM) images of the Parylene C micropore-arrayed filtration membrane. S6

Figure S2. Viability of cells released from the micropore-arrayed filtration membrane and control group cells, assessed via a live/dead staining kit. S7

Figure S3. Representative image shows lung tissue from a lung cancer rat model euthanized on day 15. S8

Figure S4. Evaluation of the metastatic potential in PC9 and PC9-CTC cells. S9

Figure S5. Immunofluorescence images of PC9-CTC spheroids cultured *in vitro*. S10

Figure S6. Representative fluorescence images of WBCs (CD45+) captured from the peripheral blood of five patients with benign pulmonary nodules. S11

Figure S7. Results of 200 permutation tests of the PLS-DA models for pairwise comparisons between PC9-CTC, Bom, and Brm cell lines. S12

Figure S8. KEGG enrichment analysis of differential metabolites for pairwise comparisons between PC9-CTC, Bom, and Brm cell lines. S13

Figure S9. The heterogeneity of CTCs and their potential utility in identifying metastatic risk in lung cancer patients. S14

**Supporting Tables:**

Table S1. Clinical information of lung cancer patients in the CTC sorting and capture platform test cohort. S15

Table S2. Metabolites identified in cell lines at single-cell level in positive ion mode. S16

Table S3. Clinical information of lung cancer patients in the training cohort. S37

Table S4. Clinical characteristics and biomarker levels of lung cancer patients in the training cohort. S38

Table S5. Total CTC count, CTC1 count, CTC2 count and CTC3 count of each patient in the training cohort.  S39

Table S6. Proportions of CTCs across different patient cohorts within each cluster. S40

Table S7. Univariate logistic regression analyses of CTC counts, clinical characteristics and biomarker levels with brain metastatic risk of lung cancer in the training cohort.  S41

Table S8. Univariate and multivariate logistic regression analyses of CTC counts, clinical characteristics and biomarker levels with bone metastatic risk of lung cancer in the training cohort. S42

Experimental Section

*Supplementary Method 1. Short-term Viability Analysis of Released Cells:* Short-term viability assessment of released cells was performed using the Calcein-AM/PI Live/Dead Cell Double Staining Kit (Solarbio Science & Technology). For staining, 2 μL of Calcein-AM probe was added per 1 mL of cell suspension, gently mixed by pipetting, and incubated at 37°C in the dark for 20 min. Subsequently, 5 μL of PI probe was added to the stained cells and incubated at room temperature in the dark for 5 min. After fluorescent labeling, cells were centrifuged at 450*g* for 5 min to remove the staining solution. The fluorescently labeled cells were resuspended in buffer solution and imaged under an inverted fluorescence microscope. Cell counts in different fluorescence channels were recorded to calculate the proportion of live and dead cells.

*Supplementary Method 2. Transwell Invasion/Migration Assay:* To evaluate the invasion and migration capabilities of PC9-CTCs versus PC9 cells, cells were resuspended in serum-free medium at a density of 1 × 10^5^ cells/well (200 μL) and seeded into the upper chamber of Transwell inserts (Corning, New York, USA). For invasion assays, the upper chambers were pre-coated with Matrigel matrix (Corning, New York, USA). The lower chambers were filled with 500 μL of medium containing 10% FBS. After 24 and 48 h of incubation, non-migrated/non-invaded cells in the upper chambers were removed using cotton swabs. Migrated or invaded cells on the lower chamber membrane were fixed with 4% paraformaldehyde, stained with 0.1% crystal violet, and imaged under a microscope. Cell counts were quantified using ImageJ software.

*Supplementary Method 3. 5-Ethynyl-2′-Deoxyuridine (EdU) Assay:* To assess the proliferative capacity of PC9-CTCs and PC9 cells, a commercial EdU assay kit (Beyotime Biotechnology, Shanghai, China) was used. Briefly, cells seeded in 6-well plates were cultured for 48 h, followed by incubation with 10 μM EdU for 2 h. Cells were fixed with 4% paraformaldehyde, permeabilized with 0.3% Triton X-100, and subjected to a click reaction with Alexa Fluor 488-conjugated azide to label proliferating cells. Nuclei were counterstained with Hoechst 33342. Fluorescence microscopy was used to visualize EdU-positive cells, and the percentage of proliferating cells was calculated.

*Supplementary Method 4. Construction of Lung Cancer Organoid Models:* The lung cancer organoid model constructed using 3D thermal bubble-based bioprinting technology was prepared as follows: A 100 μL (4×10⁴ cells/mL) PBS suspension containing PC9-CTC cells was separately loaded into the printhead. Under room temperature conditions, specific deposition coordinates were identified using the bioprinter’s integrated microscope, followed by cell deposition via view-guided printing mode onto a PDMS chip pre-coated with a Matrigel matrix (Corning, New York, USA) substrate. Post-printing, the chip was transferred to a 50 mm culture dish. Corresponding cell culture medium was gently infused using a pipette, and organoids were cultured in RPMI 1640 medium (Meilunbio, Dalian, China) supplemented with 1% fetal bovine serum (FBS) and 1% penicillin/streptomycin (P/S). All organoids were maintained in a humidified incubator at 37°C with 5% CO₂.

*Data Processing and Statistical Analysis:* Raw single-cell mass spectrometry (MS) data, acquired using Xcalibur software (v2.2, Thermo Fisher Scientific, San Jose, CA, USA), were exported as CSV files containing m/z values, intensity, and signal-to-noise ratio (S/N). Custom scripts developed in R or Python were utilized to process these data. Phosphorylcholine (m/z 184.0733) served as the marker ions for identifying single-cell events, enabling the extraction of all associated ion signals generated from ionized cellular contents. Noise filtering was applied by removing features with S/N ≤ 3. Subsequently, peak alignment across all singe-cell datasets was performed using the custom R script, employing a 25% detection rate threshold and total ion current (TIC) normalization.

Tentative metabolite identification was achieved by matching accurate masses (mass error tolerance < 5 ppm) against a custom-built database and the Human Metabolome Database (HMDB; https://hmdb.ca/). Dimensionality reduction of the complex metabolic profiles was performed using the t-distributed stochastic neighbor embedding (t-SNE) and Uniform Manifold Approximation and Projection (UMAP) algorithms, projecting data onto a two-dimensional plane for visualization. Pairwise partial least squares discriminant analysis (PLS-DA) was conducted using SIMCA-P software (v14.1, Umetrics, Umeå, Sweden). Volcano plots (generated with GraphPad Prism v9.5) and heatmaps (constructed using MetaboAnalyst 6.0; https://www.metaboanalyst.ca/) were employed to visualize differential metabolite expression patterns between groups. Pathway enrichment analysis based on the Kyoto Encyclopedia of Genes and Genomes (KEGG) was performed via the MetaboAnalyst platform.

The statistical significance of metabolic profile differences between experimental groups was assessed using the Mann-Whitney *U* test (GraphPad Prism version 9.5, GraphPad Software, Boston, MA, USA) with a *p*-value < 0.05 denoting statistical significance. Analysis of variance (ANOVA) was applied to examine the relationship between circulating tumor cell (CTC) subgroup counts and metastatic status in lung cancer patients. Student’s *t*-test was utilized to assess: (i) the correlation between total CTC counts and lung cancer pathological types, and (ii) the associations of total CTC counts and CTC subgroup numbers with brain or bone metastasis. Method performance was evaluated using the area under the curve (AUC) of receiver operating characteristic (ROC) curves to determine predictive capability for metastasis risk. The optimal cutoff value was determined by maximizing Youden’s index (J = sensitivity + specificity - 1) derived from the ROC curve. Univariate logistic regression identified factors associated with metastasis risk. Variables yielding *p*-values < 0.05 in univariate analysis were included in the multivariate logistic regression model.

All statistical analyses were performed using GraphPad Prism version 9.5. A two-sided *p*-value < 0.05 was considered statistically significant throughout the study.

**Supporting Figures:**


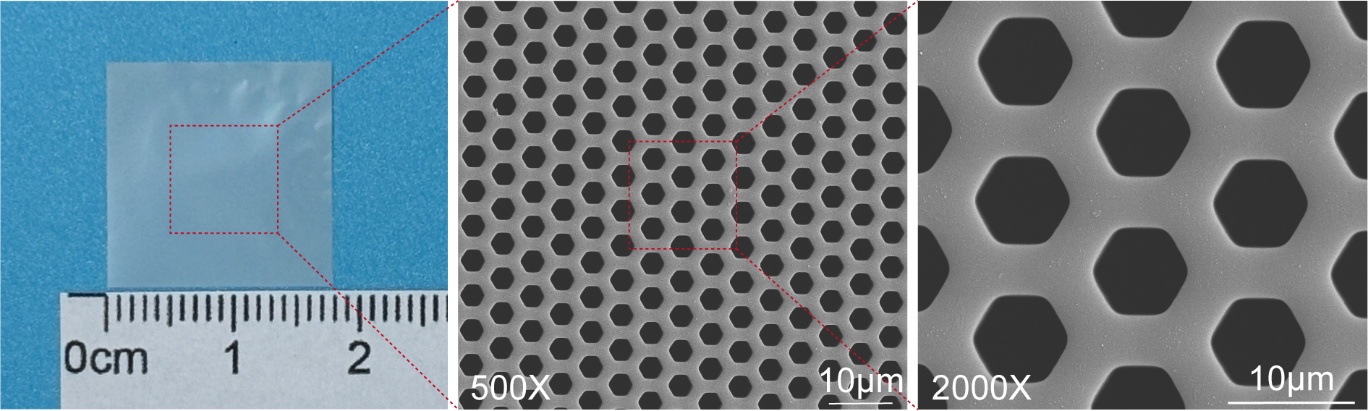


**Figure S1.** The optical and scanning electron microscopy (SEM) images of the Parylene C micropore-arrayed filtration membrane.


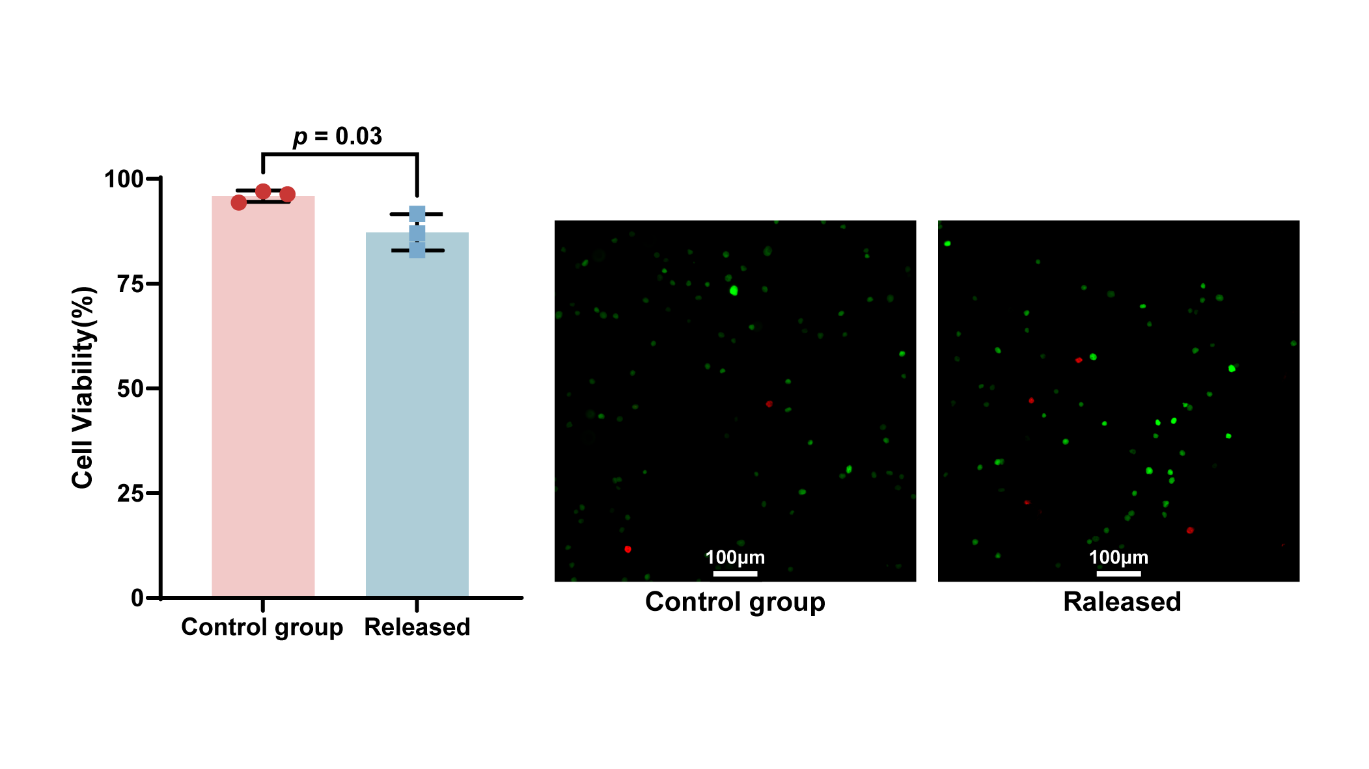


**Figure S2.** Viability of cells released from the micropore-arrayed filtration membrane and control group cells, assessed via a live/dead staining kit (green for live cells and red for dead cells). Values represent mean ± SD (*n* = 3 independent experiments). Statistical analysis was performed using the two-tailed Student’s *t*-test.


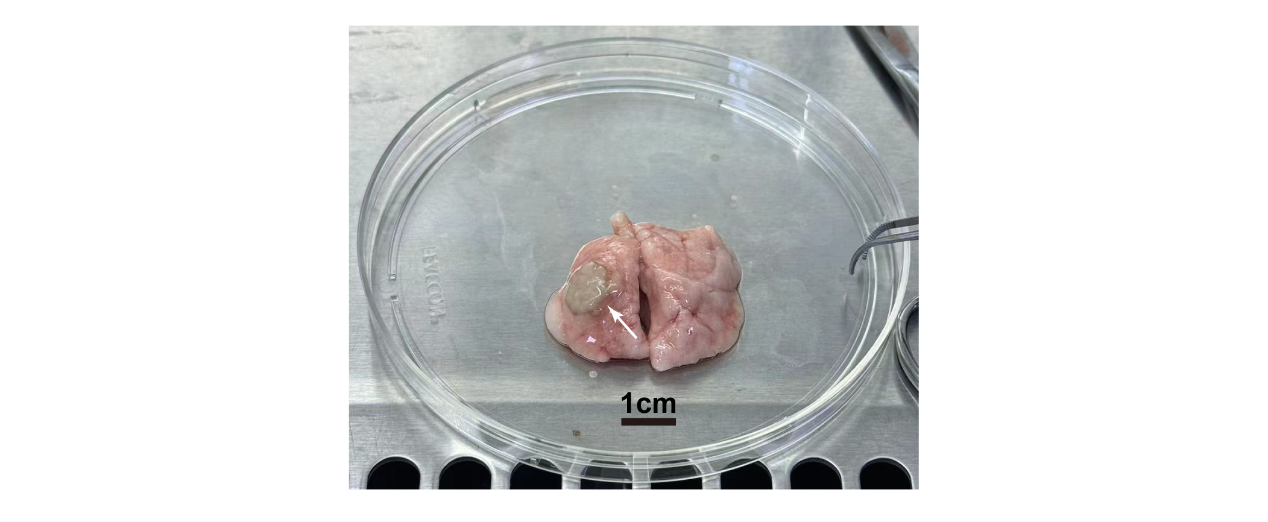


**Figure S3.** Representative image shows lung tissue from a lung cancer rat model euthanized on day 15. The sample was placed on a 10 cm Petri dish lid for scale reference. The orthotopic lung tumor measured 1.3 × 0.8 cm (long × short diameter), with an estimated volume of 0.43 cm³ (calculated using the ellipsoid formula: V = π/6 × long diameter × short diameter²). The orthotopic lung tumor is indicated by white arrow.


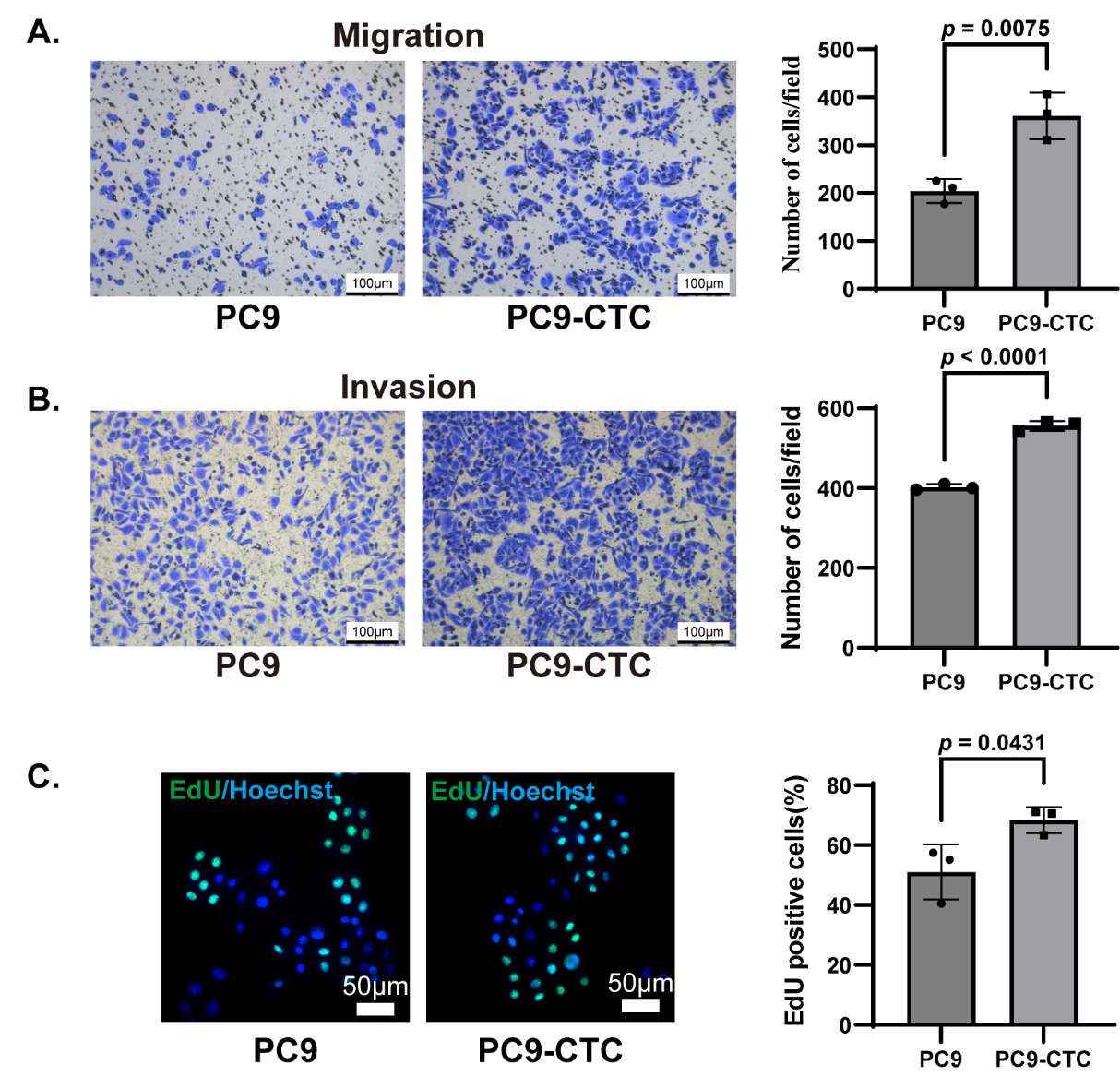


**Figure S4.** Evaluation of the metastatic potential in PC9 and PC9-CTC cells. **A)** Migration and **B)** Invasion capacities of PC9-CTCs (isolated from lung cancer rat models) compared to parental PC9 cells, evaluated by Transwell assays. Migrated/invaded cells were quantified by crystal violet staining. **C)** Proliferation of PC9-CTCs vs. parental PC9 cells analyzed via EdU incorporation assay. **Statistical analysis:** Data in **A**, **B and C** are presented as mean ± SD. Significance was determined by two-tailed Student’s *t*-test. Experiments in **A, B, and C** were repeated three times independently (*n* = 3).


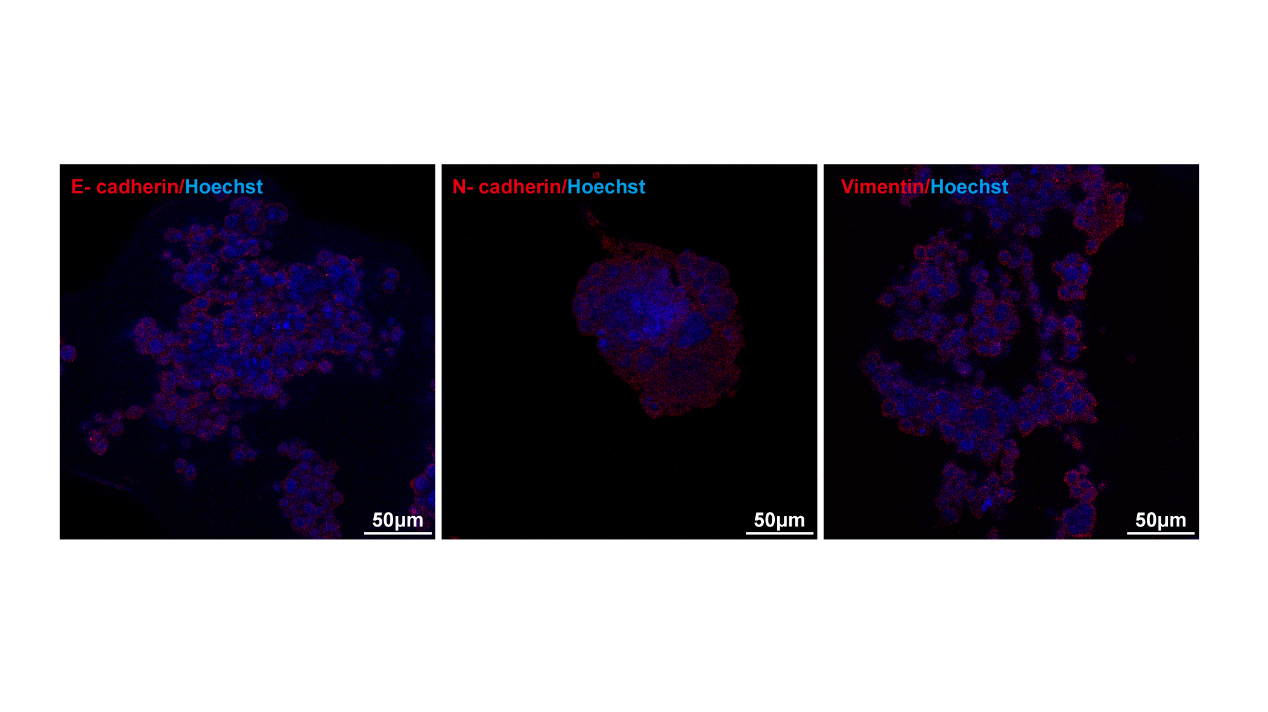


**Figure S5.** Immunofluorescence images of PC9-CTC spheroids cultured *in vitro*.


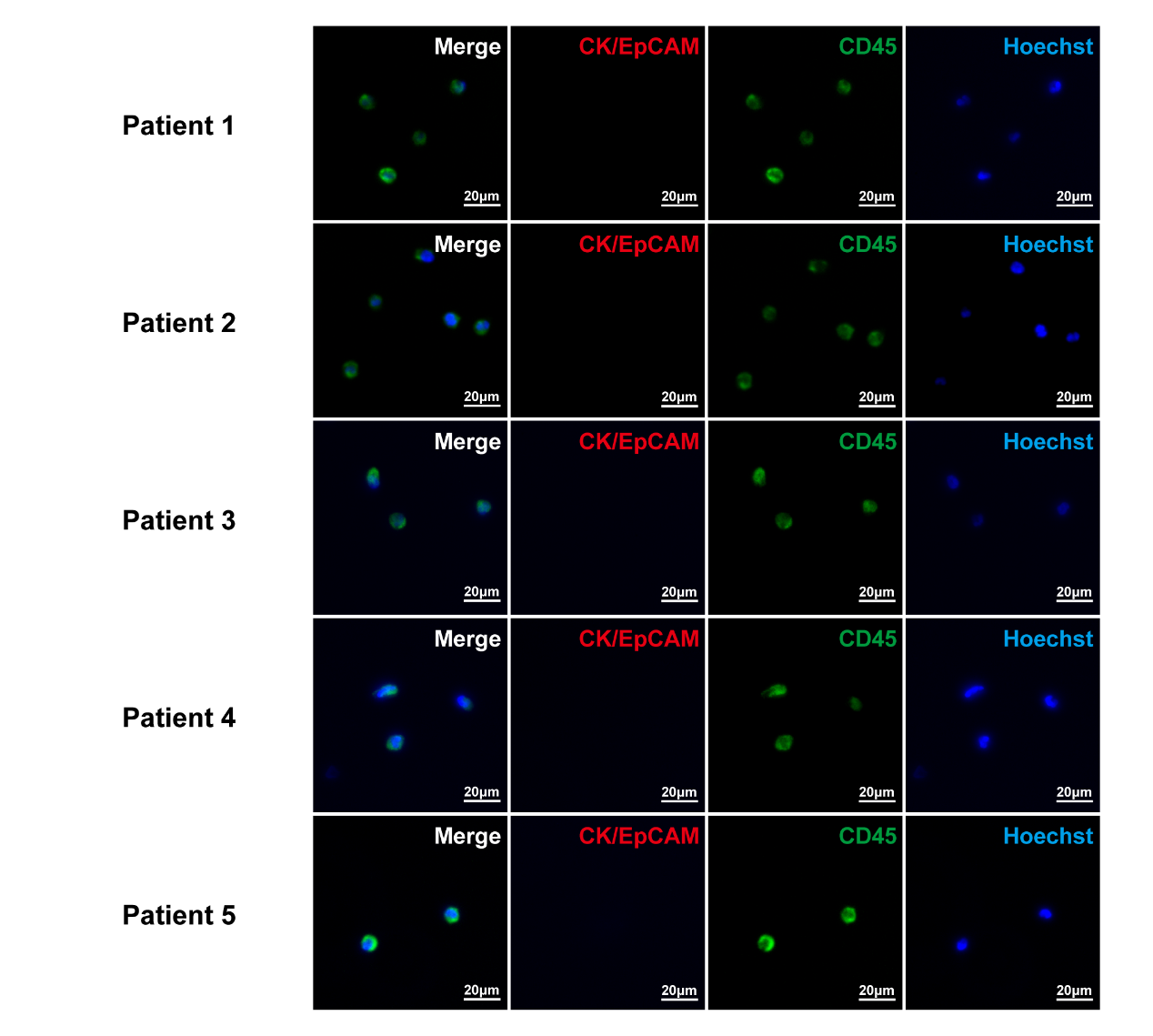


**Figure S6.** Representative fluorescence images of WBCs (CD45+) captured from the peripheral blood of five patients with benign pulmonary nodules.


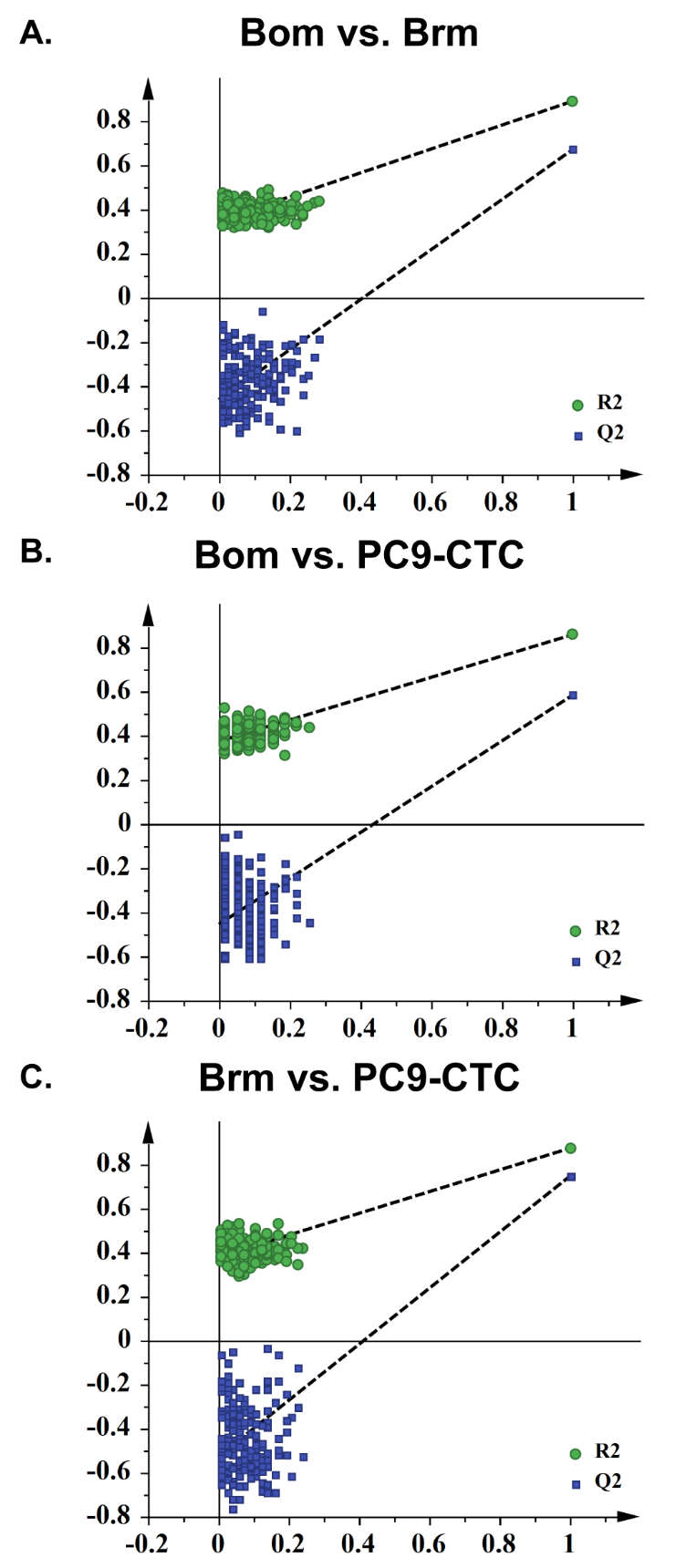


**Figure S7.** Results of 200 permutation tests of the PLS-DA models for pairwise comparisons between PC9-CTC, Bom, and Brm cell lines. **A)** Bom vs. Brm; **B)** Bom vs. PC9-CTC; **C)** Brm vs. PC9-CTC.


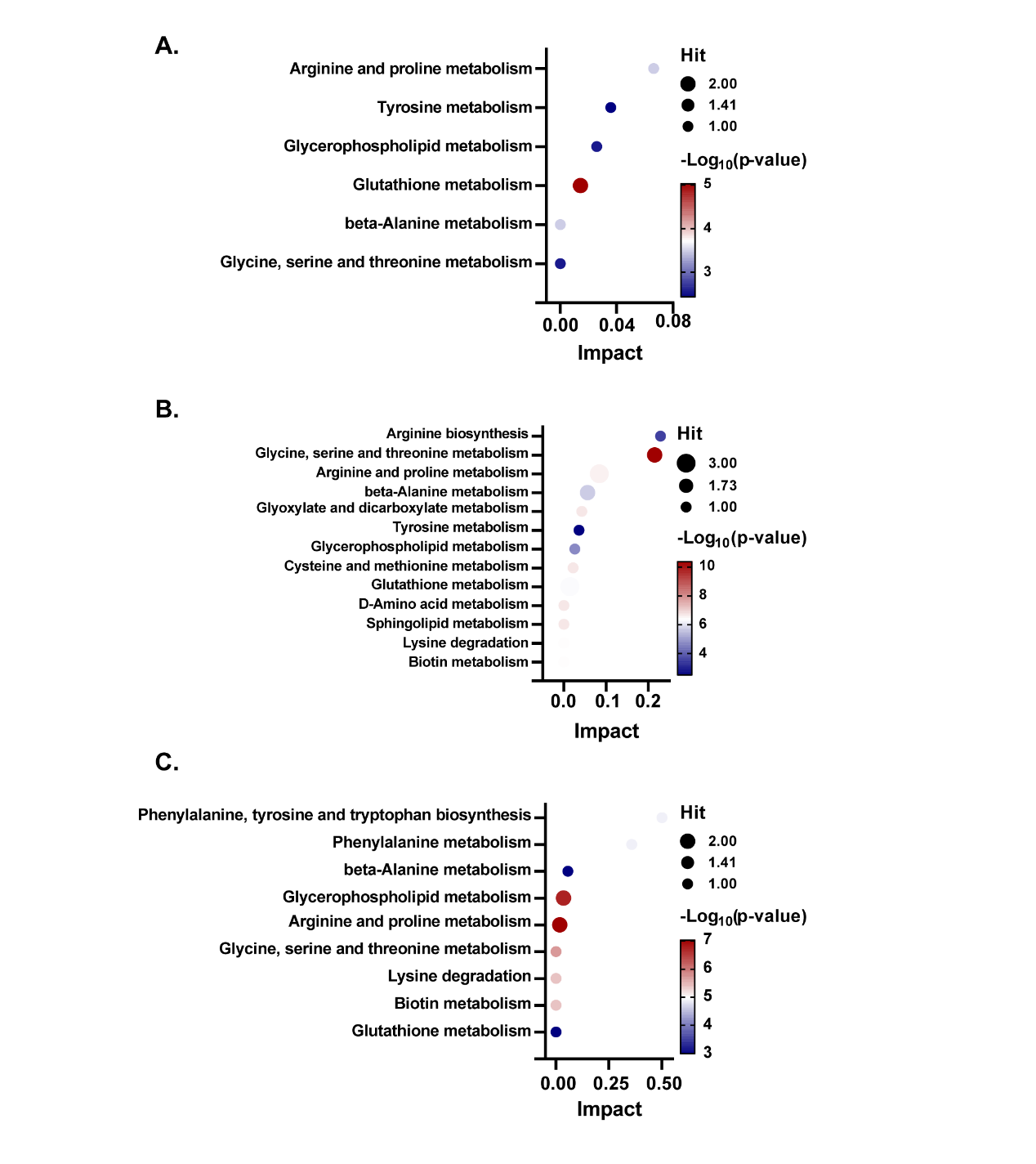


**Figure S8.** KEGG enrichment analysis of differential metabolites for pairwise comparisons between PC9-CTC, Bom, and Brm cell lines. **A)** Bom vs. Brm; **B)** Bom vs. PC9-CTC; **C)** Brm vs. PC9-CTC. The color of the points corresponded to the magnitude of –log10 (*p*-values) indicating the significance levels. The size of the points represented the number of metabolites associated with the corresponding pathway.


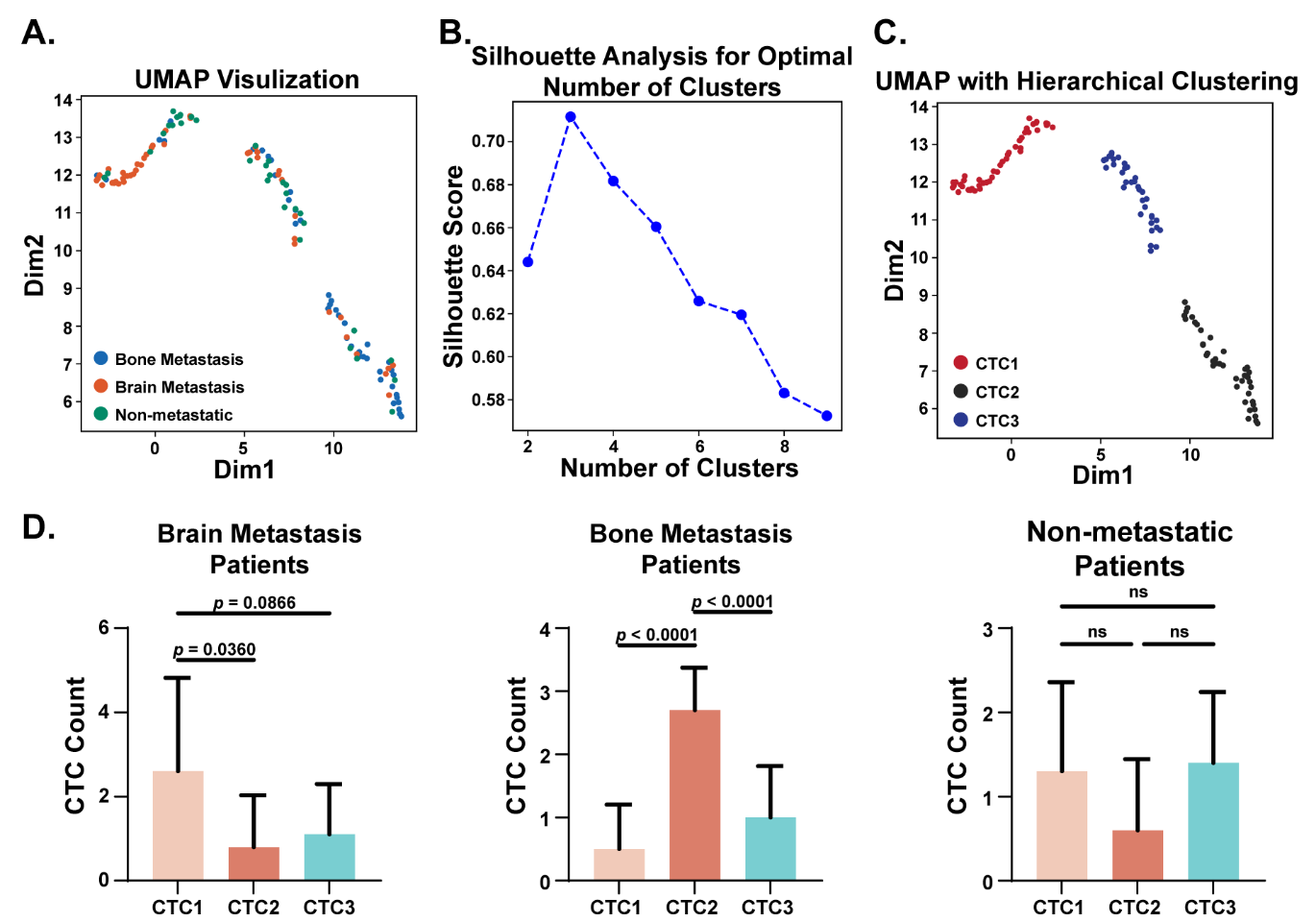


**Figure S9.** The heterogeneity of CTCs and their potential utility in identifying metastatic risk in lung cancer patients. **A)** UMAP visualization of CTCs from patients. **B)** Silhouette analysis based on dimension reduction using UMAP. **C)** UMAP visualization of CTCs labeled by hierarchical clustering. **D)** Distributions of CTC subgroups in patients. Statistical analysis: Data in **D** is presented as mean ± SD (*n* = 10). Significance was determined by one-way Analysis of Variance.

**Supporting Tables:**

**Table S1.** Clinical information of lung cancer patients in the CTC sorting and capture platform test cohort.

| **Patient No.** | **Age** | **Sex** | **Stage** | **Pathology type** | **Metastatic sites** | **CTC count** |
| --- | --- | --- | --- | --- | --- | --- |
| 1 | 71 | M | Ⅳ | Squamous cell carcinoma | Lung | 2 |
| 2 | 42 | M | Ⅳ | Adenocarcinoma | Lung, bone | 7 |
| 3 | 72 | M | Ⅳ | Adenocarcinoma | Brain | 0 |
| 4 | 58 | M | Ⅲb | Squamous cell carcinoma | None | 0 |
| 5 | 70 | F | Ⅳ | Adenocarcinoma | Lung, bone | 8 |
| 6 | 81 | F | Ⅳ | Adenocarcinoma | Lung, peritoneum | 6 |
| 7 | 62 | F | Ⅳ | Adenocarcinoma | Brain, bone | 4 |
| 8 | 72 | F | Ⅲc | Adenocarcinoma | None | 0 |
| 9 | 67 | M | Ⅳ | Adenocarcinoma | Bone, pleura | 1 |
| 10 | 61 | F | Ⅳ | Adenocarcinoma | Lung, brain, bone, pleura | 1 |
| 11 | 66 | M | Ⅲb | Adenocarcinoma | None | 4 |
| 12 | 57 | F | Ⅳ | Adenocarcinoma | Lung, pleura | 9 |
| 13 | 63 | F | Ⅳ | Squamous cell carcinoma | Lung, brain | 0 |
| 14 | 68 | M | Ⅲb | Squamous cell carcinoma | None | 5 |
| 15 | 54 | F | Ⅳ | Adenocarcinoma | Lung | 1 |
| 16 | 77 | F | Ⅳ | Adenocarcinoma | Bone | 3 |
| 17 | 51 | M | Ⅳb | Adenocarcinoma | Lung, bone | 6 |
| 18 | 64 | M | Ⅳ | Squamous cell carcinoma | Brain, bone, adrenal gland | 4 |
| 19 | 54 | M | Ⅲb | Adenocarcinoma | None | 0 |
| 20 | 67 | F | Ⅳb | Adenocarcinoma | Lung, bone, pleura | 15 |
| 21 | 70 | M | Ⅲb | Adenocarcinoma | None | 2 |
| 22 | 60 | F | Ⅳ | Adenocarcinoma | Lung, pleura, peritoneum | 3 |
| 23 | 71 | F | Ⅳ | Adenocarcinoma | Lung, brain, bone, liver, pleura | 11 |
| 24 | 78 | F | Ⅲc | Squamous cell carcinoma | None | 0 |
| 25 | 66 | F | Ⅳ | Adenocarcinoma | Brain, bone | 7 |
| 26 | 59 | F | Ⅲb | Adenocarcinoma | None | 2 |
| 27 | 64 | M | Ⅳ | Adenocarcinoma | Lung, bone, liver, adrenal gland | 7 |
| 28 | 79 | M | Ⅳ | Squamous cell carcinoma | Lung | 2 |
| 29 | 74 | M | Ⅲa | Squamous cell carcinoma | None | 5 |
| 30 | 68 | F | Ⅲa | Adenocarcinoma | None | 3 |
| 31 | 54 | F | Ⅳ | Adenocarcinoma | Lung, brain, bone, liver | 4 |

**Table S2.** Metabolites identified in cell lines at single-cell level in positive ion mode.

| **No.** | **Metabolite name** | **Database mass, m/z** | **Adduct Ion** | **Measured mass, m/z** | **Error, ppm** | **LC-MS MS2 check** |
| --- | --- | --- | --- | --- | --- | --- |
| 1 | 4-Aminobutyraldehyde | 70.0657 | M+H-H2O | 70.06569 | 0.09 |  |
| 2 | Butyric acid | 71.0497 | M+H-H2O | 71.0497 | 0.07 |  |
|  |  | 89.0597 | M+H | 89.0601 | 4.44 |  |
|  |  | 106.0862 | M+NH4 | 106.0865 | 2.47 |  |
|  |  | 111.0416 | M+Na | 111.0418 | 2.06 |  |
| 3 | Alanine | 72.045 | M+H-H2O | 72.04494 | 0.77 |  |
|  |  | 90.055 | M+H | 90.05535 | 3.93 |  |
|  |  | 112.0369 | M+Na | 112.037 | 1.18 |  |
| 4 | Lactic acid | 73.029 | M+H-H2O | 73.02896 | 0.54 |  |
|  |  | 91.039 | M+H | 91.03934 | 3.7 |  |
|  |  | 108.0655 | M+NH4 | 108.0657 | 1.68 |  |
|  |  | 113.0209 | M+Na | 113.021 | 1.29 |  |
| 5 | 1,3-Butanediol | 73.0654 | M+H-H2O | 73.06532 | 1.12 |  |
| 6 | Butylamine | 74.0966 | M+H | 74.09695 | 4.78 | √ |
| 7 | 2-Propene-1-thiol | 75.0263 | M+H | 75.02661 | 4.07 |  |
| 8 | Glycerol | 75.0446 | M+H-H2O | 75.04458 | 0.24 |  |
|  |  | 93.0546 | M+H | 93.05495 | 3.8 |  |
|  |  | 110.0811 | M+NH4 | 110.0813 | 1.99 |  |
|  |  | 115.0365 | M+Na | 115.0367 | 2.06 |  |
| 9 | 4-Hydroxymethylpyrazole | 81.0453 | M+H-H2O | 81.04519 | 1.35 |  |
| 10 | Pyrazine | 81.053 | M+H | 81.0533 | 3.76 |  |
| 11 | 2-Hexenal | 81.0705 | M+H-H2O | 81.07035 | 1.87 |  |
|  |  | 99.0805 | M+H | 99.08073 | 2.31 |  |
|  |  | 121.0624 | M+Na | 121.0625 | 0.69 |  |
| 12 | Glutaral | 83.0497 | M+H-H2O | 83.04959 | 1.3 |  |
|  |  | 101.0597 | M+H | 101.06 | 2.68 |  |
|  |  | 123.0416 | M+Na | 123.0417 | 0.53 |  |
| 13 | 4-Methylpentanal | 83.0861 | M+H-H2O | 83.08598 | 1.46 |  |
|  |  | 123.078 | M+Na | 123.0781 | 0.97 |  |
| 14 | 2-Azetidinecarboxylic acid | 84.045 | M+H-H2O | 84.04483 | 1.97 |  |
| 15 | 2-Ketobutyric acid | 85.029 | M+H-H2O | 85.02884 | 1.94 |  |
| 16 | 2-Pentenal | 85.0648 | M+H | 85.06522 | 4.97 |  |
|  |  | 102.0913 | M+NH4 | 102.0916 | 2.59 |  |
|  |  | 107.0467 | M+Na | 107.0471 | 4.09 |  |
| 17 | 3-Hexanol | 85.1018 | M+H-H2O | 85.10161 | 2.24 |  |
| 18 | gamma-Aminobutyric acid | 86.0606 | M+H-H2O | 86.06046 | 1.64 |  |
|  |  | 104.0706 | M+H | 104.0708 | 2.17 |  |
| 19 | Crotonic acid | 87.0441 | M+H | 87.04446 | 4.15 | √ |
| 20 | 2,3-Diaminopropionic acid | 87.0559 | M+H-H2O | 87.05571 | 2.2 |  |
| 21 | Pentanal | 87.0805 | M+H | 87.08085 | 3.98 |  |
|  |  | 109.0624 | M+Na | 109.0628 | 4.02 |  |
| 22 | Serine | 88.0399 | M+H-H2O | 88.03971 | 2.2 |  |
|  |  | 106.0499 | M+H | 106.0501 | 1.91 | √ |
| 23 | 2-Amino-2-methyl-1,3-propanediol | 88.0757 | M+H-H2O | 88.07608 | 4.37 | √ |
| 24 | 3-Methylbutylamine | 88.1125 | M+H | 88.11248 | 0.23 | √ |
| 25 | Pyruvic acid | 89.0233 | M+H | 89.02373 | 4.79 |  |
| 26 | Methyl-1-butanol | 89.0961 | M+H | 89.0965 | 4.52 |  |
| 27 | Putrescine | 89.1073 | M+H | 89.10772 | 4.75 | √ |
| 28 | 1,3-Dihydroxyacetone | 91.0573 | M+H | 91.05775 | 4.9 |  |
| 29 | Butanediol | 91.0754 | M+H | 91.07572 | 3.48 |  |
|  |  | 108.1019 | M+NH4 | 108.1021 | 1.5 |  |
|  |  | 113.0573 | M+Na | 113.0575 | 1.78 |  |
| 30 | 3-Aminopropionaldehyde | 91.0866 | M+NH4 | 91.08696 | 3.95 |  |
|  |  | 96.042 | M+Na | 96.0423 | 3.15 |  |
| 31 | Lactaldehyde | 92.0706 | M+NH4 | 92.07096 | 3.89 |  |
|  |  | 97.026 | M+Na | 97.0263 | 3.08 |  |
| 32 | 2-(Methylthio)ethanol | 93.0369 | M+H | 93.03697 | 0.75 |  |
| 33 | Aniline | 94.0651 | M+H | 94.06546 | 3.85 | √ |
| 34 | Butanal | 95.0467 | M+Na | 95.04702 | 3.4 |  |
| 35 | 2,4-Hexadienoic acid | 95.0497 | M+H-H2O | 95.04947 | 2.43 |  |
| 36 | 2-Aminopyridine | 95.0603 | M+H | 95.0607 | 4.23 |  |
| 37 | 1,2,3,6-Tetrahydrobenzylalcohol | 95.0855 | M+H-H2O | 95.08585 | 3.68 | √ |
| 38 | 4-Hydroxypyridine | 96.0443 | M+H | 96.04471 | 4.3 |  |
| 39 | Propionic acid | 97.0283 | M+Na | 97.02871 | 4.21 |  |
| 40 | 4-Hydroxyhexenal | 97.0648 | M+H-H2O | 97.0651 | 3.06 | √ |
| 41 | 1-Methylcyclohexanol | 97.101 | M+H-H2O | 97.10148 | 4.91 |  |
| 42 | Proline | 98.0606 | M+H-H2O | 98.06033 | 2.75 |  |
|  |  | 116.0706 | M+H | 116.0707 | 0.8 | √ |
|  |  | 138.0525 | M+Na | 138.0523 | 1.44 |  |
| 43 | N-(3-Methylbutyl)acetamide | 98.097 | M+H-H2O | 98.09671 | 2.91 |  |
| 44 | alpha-Ketoisovaleric acid | 99.0446 | M+H-H2O | 99.04433 | 2.74 |  |
|  |  | 139.0365 | M+Na | 139.0363 | 1.33 |  |
| 45 | 2-Heptanol | 99.1174 | M+H-H2O | 99.11712 | 2.86 |  |
| 46 | Acetylglycine | 100.0399 | M+H-H2O | 100.0396 | 3.29 |  |
|  |  | 118.0499 | M+H | 118.0499 | 0.18 |  |
|  |  | 140.0318 | M+Na | 140.0316 | 1.49 |  |
| 47 | 2-Piperidone | 100.0757 | M+H | 100.076 | 2.51 |  |
| 48 | Cyclohexanamine | 100.1119 | M+H | 100.1123 | 4.41 |  |
| 49 | Succinic acid | 101.0239 | M+H-H2O | 101.0236 | 3.11 |  |
|  |  | 136.0604 | M+NH4 | 136.0603 | 0.66 |  |
|  |  | 141.0158 | M+Na | 141.0156 | 1.24 |  |
| 50 | Methylpentanal | 101.0961 | M+H | 101.0963 | 2.25 |  |
|  |  | 118.1226 | M+NH4 | 118.1227 | 0.81 |  |
| 51 | Hexylamine | 102.1284 | M+H | 102.128 | 4.3 | √ |
| 52 | Succinic acid semialdehyde | 103.039 | M+H | 103.0392 | 1.96 |  |
|  |  | 125.0209 | M+Na | 125.0209 | 0.13 |  |
| 53 | 3-Amino-2-oxazolidinone | 103.0504 | M+H | 103.0504 | 0.41 | √ |
| 54 | Pentanoic acid | 103.0754 | M+H | 103.0756 | 1.78 |  |
| 55 | Choline | 104.1069 | M | 104.1072 | 3.14 |  |
| 56 | 2-Cyanopyridine | 105.0032 | M+H | 105.0035 | 2.97 |  |
| 57 | Hydroxybutyric acid | 105.0546 | M+H | 105.0549 | 2.45 |  |
|  |  | 122.0811 | M+NH4 | 122.0812 | 0.76 |  |
| 58 | Diaminopropionic acid | 105.0659 | M+H | 105.0661 | 1.74 |  |
| 59 | 3-Methylbenzyl alcolhol | 105.0706 | M+H-H2O | 105.0701 | 4.71 |  |
| 60 | 3-Methylthiopropylamine | 106.065 | M+H | 106.0654 | 3.31 |  |
| 61 | 4-Hydroxy-2-butenoic acid gamma-lactone | 107.0103 | M+Na | 107.0106 | 2.36 |  |
| 62 | 3-Hydroxybenzyl alcohol | 107.0496 | M+H-H2O | 107.0493 | 2.52 | √ |
| 63 | Diethylene glycol | 107.0701 | M+H | 107.0705 | 3.37 |  |
| 64 | 1-Ethynylcyclohexanamine | 107.0855 | M+H-NH3 | 107.0857 | 1.93 | √ |
| 65 | Pyrrolidinone | 108.042 | M+Na | 108.0424 | 3.42 |  |
| 66 | Benzylamine | 108.0806 | M+H | 108.0809 | 3.13 |  |
| 67 | gamma-Butyrolactone | 109.026 | M+Na | 109.0262 | 1.49 |  |
| 68 | 2-Ethylpyrazine | 109.076 | M+H | 109.0762 | 1.64 |  |
| 69 | Phenylhydrazine | 109.101 | M+H | 109.1013 | 3.02 |  |
| 70 | Aminobutyraldehyde | 110.0576 | M+Na | 110.0578 | 2.13 |  |
| 71 | 4-Aminophenol | 110.0599 | M+H | 110.0602 | 2.32 |  |
| 72 | 5-Methyl-2-furancarboxaldehyde | 111.0441 | M+H | 111.0442 | 0.82 |  |
| 73 | Pipecolic acid | 112.0763 | M+H-H2O | 112.0758 | 4.23 |  |
|  |  | 130.0863 | M+H | 130.0862 | 0.98 | √ |
| 74 | Histamine | 112.0836 | M+H | 112.0839 | 2.93 |  |
| 75 | 2-Furancarboxylic acid | 113.0231 | M+H | 113.0232 | 1.23 |  |
| 76 | Sorbic acid | 113.0593 | M+H | 113.0598 | 4.55 | √ |
| 77 | Heptenal | 113.0961 | M+H | 113.0962 | 1.03 |  |
|  |  | 130.1226 | M+NH4 | 130.1225 | 0.52 |  |
| 78 | N-Acetylputrescine | 113.1079 | M+H-H2O | 113.1075 | 3.83 |  |
|  |  | 131.118 | M+H | 131.1178 | 1.79 |  |
| 79 | Pyrroline-5-carboxylic acid | 114.055 | M+H | 114.0551 | 0.54 |  |
| 80 | Creatinine | 114.0681 | M+H | 114.0677 | 3.66 |  |
| 81 | Leucine | 114.0912 | M+H-H2O | 114.0914 | 2.16 |  |
|  |  | 154.0838 | M+Na | 154.0835 | 1.9 |  |
| 82 | Glutaric acid | 115.0388 | M+H-H2O | 115.0391 | 2.23 |  |
|  |  | 133.0496 | M+H | 133.0494 | 1.42 |  |
|  |  | 150.0761 | M+NH4 | 150.0759 | 1.26 |  |
|  |  | 155.0315 | M+Na | 155.0312 | 1.9 |  |
| 83 | Dihydrouracil | 115.0502 | M+H | 115.0503 | 1.05 |  |
| 84 | Hexanolactone | 115.0754 | M+H | 115.0755 | 0.47 |  |
| 85 | Ketoisovaleric acid | 117.0546 | M+H | 117.0547 | 0.65 |  |
| 86 | Cinnamyl alcohol | 117.0705 | M+H-H2O | 117.0699 | 4.81 |  |
|  |  | 135.0805 | M+H | 135.0803 | 1.23 |  |
| 87 | Caproic acid | 117.091 | M+H | 117.0911 | 0.55 |  |
|  |  | 134.1175 | M+NH4 | 134.1174 | 0.52 |  |
|  |  | 139.0729 | M+Na | 139.0727 | 1.45 |  |
| 88 | Indole | 118.0648 | M+H | 118.0651 | 2.93 | √ |
| 89 | Valine | 118.0863 | M+H | 118.0863 | 0.01 | √ |
| 90 | N-Methylnicotinamide | 119.061 | M+H-H2O | 119.0604 | 4.78 |  |
| 91 | 2-Methyl-3-hydroxybutyric acid | 119.0703 | M+H | 119.0703 | 0.04 |  |
|  |  | 136.0968 | M+NH4 | 136.0967 | 1.04 |  |
|  |  | 141.0522 | M+Na | 141.0519 | 2.22 |  |
| 92 | Diaminobutyric acid | 119.0815 | M+H | 119.0816 | 0.64 |  |
| 93 | beta-N-Methylaminoalanine | 119.0854 | M+H | 119.0856 | 1.32 |  |
| 94 | Hexanediol | 119.1067 | M+H | 119.1067 | 0.09 |  |
| 95 | Threonine | 120.0655 | M+H | 120.0655 | 0.29 |  |
|  |  | 142.0474 | M+Na | 142.0472 | 1.24 |  |
| 96 | Methyl butyrate | 120.1019 | M+NH4 | 120.1019 | 0.31 |  |
| 97 | Salicylic acid | 121.0284 | M+H-H2O | 121.0284 | 0.31 | √ |
|  |  | 139.0388 | M+H | 139.0389 | 0.61 |  |
| 98 | Tolualdehyde | 121.0653 | M+H | 121.0648 | 4.27 | √ |
| 99 | N-Ethylaniline | 122.0964 | M+H | 122.0964 | 0.32 | √ |
| 100 | 4-Hydroxybenzaldehyde | 123.0445 | M+H | 123.0441 | 3.48 | √ |
| 101 | Niacinamide | 123.0553 | M+H | 123.0553 | 0.11 | √ |
| 102 | 2,5-Dimethylphenol | 123.0805 | M+H | 123.0804 | 0.57 |  |
| 103 | Nicotinic acid | 124.0393 | M+H | 124.0393 | 0.29 |  |
| 104 | Anisidine | 124.0757 | M+H | 124.0757 | 0.04 |  |
| 105 | Ethyl propionate | 125.0573 | M+Na | 125.0573 | 0.02 |  |
| 106 | 3-Hydroxyvalproic acid | 125.0961 | M+H-2H2O | 125.0961 | 0.24 | √ |
| 107 | Pyruvatoxime | 126.0161 | M+Na | 126.0161 | 0.39 |  |
| 108 | Taurine | 126.0219 | M+H | 126.0219 | 0.32 |  |
| 109 | 2-Amino-3-methylenehexanoic acid | 126.0919 | M+H-H2O | 126.0913 | 4.38 |  |
| 110 | 2-Hydroxybutyric acid | 127.0365 | M+Na | 127.0365 | 0.26 |  |
| 111 | Hydroxycyclohexylcarboxylic acid | 127.0758 | M+H-H2O | 127.0753 | 3.98 |  |
| 112 | 3,3-Dimethylcyclohexanone | 127.1117 | M+H | 127.1117 | 0.17 | √ |
| 113 | Isoguvacine | 128.0706 | M+H | 128.0705 | 0.48 |  |
| 114 | N-Cyclohexylformamide | 128.107 | M+H | 128.1069 | 0.56 |  |
| 115 | Adipic acid | 129.0521 | M+H-H2O | 129.0521 | 0.03 |  |
| 116 | Dihydrothymine | 129.0659 | M+H | 129.0658 | 1.06 |  |
| 117 | 2-Piperidinecarboxamide | 129.1022 | M+H | 129.1021 | 0.81 | √ |
| 118 | 2-Octanone | 129.1274 | M+H | 129.1273 | 0.81 |  |
| 119 | N,N-Dimethyl-3-piperidinamine | 129.1386 | M+H | 129.1385 | 0.6 | √ |
| 120 | Pyroglutamic acid | 130.0499 | M+H | 130.0498 | 0.62 | √ |
|  |  | 152.0318 | M+Na | 152.0316 | 1.13 |  |
| 121 | N-Octylamine | 130.159 | M+H | 130.1589 | 0.59 | √ |
| 122 | Ketoleucine | 131.0703 | M+H | 131.0702 | 0.74 |  |
|  |  | 153.0522 | M+Na | 153.0518 | 2.62 |  |
| 123 | Ethyl isovalerate | 131.1067 | M+H | 131.1066 | 1.07 |  |
|  |  | 148.1332 | M+NH4 | 148.133 | 1.46 |  |
| 124 | N-Acetyl-alanine | 132.0655 | M+H | 132.0654 | 0.49 |  |
| 125 | Creatine | 132.0768 | M+H | 132.0766 | 1.3 |  |
|  |  | 154.0587 | M+Na | 154.0585 | 1.16 |  |
| 126 | Isoleucine | 132.1016 | M+H | 132.1018 | 1.6 | √ |
| 127 | Hepten-1-ol | 132.1383 | M+NH4 | 132.1382 | 0.97 |  |
| 128 | Cinnamaldehyde | 133.0648 | M+H | 133.0647 | 0.73 |  |
|  |  | 155.0467 | M+Na | 155.0466 | 0.73 |  |
| 129 | Hydroxyhexanoic acid | 133.0859 | M+H | 133.0858 | 0.62 |  |
|  |  | 150.1125 | M+NH4 | 150.1123 | 1.35 |  |
|  |  | 155.0678 | M+Na | 155.0673 | 3.34 |  |
| 130 | Aspartic acid | 134.0448 | M+H | 134.0447 | 0.94 |  |
|  |  | 156.0267 | M+Na | 156.0265 | 1.55 |  |
| 131 | 2-Amino-5-hydroxypentanoic acid | 134.0812 | M+H | 134.0811 | 1.05 |  |
| 132 | 3-Iodotyrosine | 135.0026 | M+H | 135.0027 | 0.92 |  |
| 133 | 4-Carboxypyrazole | 135.0165 | M+Na | 135.0166 | 0.63 |  |
| 134 | 1,2-Cyclohexanedione | 135.0416 | M+Na | 135.0415 | 0.84 |  |
| 135 | Phthalide | 135.0441 | M+H | 135.0439 | 1.12 |  |
|  |  | 157.026 | M+Na | 157.0258 | 1.08 |  |
| 136 | Benzothiazole | 136.0215 | M+H | 136.0214 | 0.76 | √ |
| 137 | Acetanilide | 136.073 | M+H | 136.0731 | 0.43 |  |
| 138 | Phenylacetamide | 136.0757 | M+H | 136.0756 | 0.74 |  |
| 139 | Adenine | 136.112 | M+H | 136.1119 | 0.73 |  |
|  |  | 153.0883 | M+NH4 | 153.0883 | 0.12 |  |
| 140 | 2-Butoxyethanol | 136.1332 | M+NH4 | 136.1331 | 0.99 |  |
| 141 | delta-Hexanolactone | 137.0573 | M+Na | 137.0571 | 1.54 |  |
| 142 | Methylbenzoic acid | 137.0597 | M+H | 137.0596 | 0.83 |  |
| 143 | 3-Heptanone | 137.0937 | M+Na | 137.0935 | 1.43 |  |
| 144 | Phenelzine | 137.1072 | M+H | 137.1072 | 0.15 |  |
| 145 | Nicotinamide-N-oxide | 139.0475 | M+H | 139.0475 | 0.18 |  |
| 146 | Isophorone | 139.1116 | M+H | 139.1115 | 0.47 | √ |
| 147 | Betaine | 140.0682 | M+Na | 140.0679 | 1.87 | √ |
| 148 | 1-Phenylethanol | 140.107 | M+NH4 | 140.1068 | 1.52 |  |
| 149 | 1,5-Hexanediol | 141.0886 | M+Na | 141.0884 | 1.66 |  |
| 150 | Hexadienyl acetate | 141.091 | M+H | 141.0908 | 1.41 |  |
| 151 | 2,2,6-Trimethylcyclohexanone | 141.1274 | M+H | 141.1271 | 1.82 |  |
| 152 | Hydroxybenzyl alcohol | 142.0863 | M+NH4 | 142.0861 | 1.38 |  |
| 153 | 1,5-Octadien-3-one | 142.1226 | M+NH4 | 142.1225 | 0.93 |  |
| 154 | Deoxyerythronic acid | 143.0315 | M+Na | 143.0313 | 1.44 |  |
| 155 | Phenylacetaldehyde | 143.0467 | M+Na | 143.0466 | 0.91 |  |
| 156 | 2-Octenoic acid | 143.1061 | M+H | 143.1065 | 2.45 | √ |
| 157 | Quinaldine | 144.081 | M+H | 144.0806 | 2.58 | √ |
| 158 | 1-Amlnocydohexanecarboxylic Acid | 144.1016 | M+H | 144.1017 | 0.63 | √ |
| 159 | 2-(1-Piperidyl)propan-2-ol | 144.1381 | M+H | 144.1381 | 0.09 |  |
| 160 | Erythritol | 145.0471 | M+Na | 145.0468 | 1.8 |  |
| 161 | Dimethyl fumarate | 145.0496 | M+H | 145.0494 | 1.32 |  |
| 162 | 2-Propylpentanoic acid | 145.1222 | M+H | 145.1221 | 0.81 |  |
| 163 | N-Butyrylglycine | 146.0812 | M+H | 146.081 | 1.66 |  |
| 164 | Acetylcholine | 146.1172 | M+H | 146.1173 | 0.87 | √ |
| 165 | Spermidine | 146.1652 | M+H | 146.165 | 1.72 | √ |
| 166 | Coumaric acid | 147.0442 | M+H-H2O | 147.0439 | 2.05 |  |
| 167 | Methylglutaric acid | 147.0652 | M+H | 147.065 | 1.5 |  |
|  |  | 164.0917 | M+NH4 | 164.0915 | 1 |  |
|  |  | 169.0471 | M+Na | 169.0468 | 1.94 |  |
| 168 | Glutamine | 147.0763 | M+H | 147.0766 | 2.28 |  |
| 169 | Benzylideneacetone | 147.0807 | M+H | 147.0803 | 2.9 |  |
| 170 | Methyl leucate | 147.1016 | M+H | 147.1014 | 1.6 |  |
|  |  | 164.1281 | M+NH4 | 164.1279 | 1.32 |  |
| 171 | Lysine | 147.1128 | M+H | 147.1126 | 1.29 |  |
|  |  | 164.1393 | M+NH4 | 164.1392 | 0.33 |  |
| 172 | Glutamic acid | 148.0605 | M+H | 148.0602 | 1.79 |  |
|  |  | 170.0424 | M+Na | 170.0421 | 1.92 |  |
| 173 | 2-Amino-6-hydroxyhexanoic acid | 148.0968 | M+H | 148.0966 | 1.5 |  |
| 174 | 5-Formylsalicylic acid | 149.0229 | M+H-H2O | 149.0231 | 1.56 | √ |
|  |  | 167.0332 | M+H | 167.0338 | 3.81 | √ |
| 175 | 2-Hydroxyglutaric acid | 149.0445 | M+H | 149.0447 | 1.36 |  |
| 176 | 3-(4-Hydroxyphenyl)-propionic acid | 149.0603 | M+H-H2O | 149.0597 | 3.83 | √ |
| 177 | 1-Octen-3-one | 149.0937 | M+Na | 149.0932 | 3.05 |  |
| 178 | 3-Carboxybenzadehyde | 151.0383 | M+H | 151.0389 | 4.09 | √ |
| 179 | 2,3-Heptanedione | 151.0729 | M+Na | 151.0726 | 2.2 |  |
| 180 | Triethylene glycol | 151.0964 | M+H | 151.0963 | 0.5 |  |
| 181 | Geranic acid | 151.1112 | M+H-H2O | 151.1116 | 2.38 |  |
|  |  | 169.1223 | M+H | 169.122 | 1.65 |  |
| 182 | Dopamine quinone | 152.0706 | M+H | 152.0705 | 0.89 |  |
| 183 | Dihydroxyvaleric acid | 152.0917 | M+NH4 | 152.0916 | 0.92 |  |
| 184 | Itaconic acid | 153.0158 | M+Na | 153.0156 | 1.1 |  |
| 185 | Azelaic acid | 153.091 | M+H-2H2O | 153.091 | 0.3 | √ |
|  |  | 189.1119 | M+H | 189.1118 | 0.58 |  |
| 186 | Citral | 153.1268 | M+H | 153.1272 | 2.36 | √ |
| 187 | Benzyl ethyl ether | 154.1226 | M+NH4 | 154.1224 | 1.35 |  |
| 188 | 4-Hydroperoxy-2-nonenal | 155.1067 | M+H-H2O | 155.1064 | 1.94 | √ |
| 189 | Histidine | 156.0768 | M+H | 156.0767 | 0.93 |  |
| 190 | 4-Ethoxyphenol | 156.1019 | M+NH4 | 156.1017 | 1.26 |  |
| 191 | Allantoin | 156.8776 | M+H | 156.8773 | 2.03 |  |
| 192 | 2,3-Dihydroxyvaleric acid | 157.0471 | M+Na | 157.0468 | 1.66 |  |
| 193 | 8-Hydroxy-5,6-octadienoic acid | 157.0859 | M+H | 157.086 | 0.62 |  |
|  |  | 174.1124 | M+NH4 | 174.1122 | 1.2 |  |
|  |  | 179.0678 | M+Na | 179.0674 | 2.24 |  |
| 194 | Nonalactone | 157.1223 | M+H | 157.1221 | 1.38 | √ |
| 195 | 3-Methylcrotonylglycine | 158.0812 | M+H | 158.081 | 1.45 |  |
| 196 | Citrulline | 158.093 | M+H-H2O | 158.0932 | 1.18 |  |
|  |  | 193.131 | M+NH4 | 193.1306 | 2.27 |  |
| 197 | 2,4-Dimethylquinoline | 158.0963 | M+H | 158.0964 | 0.37 |  |
| 198 | N-Isobutyl-3-methylbutanamide | 158.1539 | M+H | 158.1537 | 1.04 | √ |
| 199 | 4-Methylbenzoic acid | 159.0416 | M+Na | 159.0414 | 1.24 |  |
| 200 | Succinylacetone | 159.0652 | M+H | 159.0651 | 0.92 |  |
|  |  | 176.0917 | M+NH4 | 176.0915 | 1.29 |  |
|  |  | 181.0471 | M+Na | 181.0468 | 1.75 |  |
| 201 | 3-Methoxycyclohexanecarboxylic acid | 159.1014 | M+H | 159.1014 | 0.2 |  |
| 202 | 1-Hydroxy-3-nonanone | 159.138 | M+H | 159.1377 | 1.78 |  |
|  |  | 176.1645 | M+NH4 | 176.1643 | 1.36 |  |
| 203 | Methylbutyrylglycine | 160.0968 | M+H | 160.0966 | 1.32 |  |
| 204 | 4-aminovaleric acid betaine | 160.1332 | M+H | 160.133 | 1.39 | √ |
| 205 | 6-Methylcoumarin | 161.0597 | M+H | 161.0596 | 0.82 |  |
| 206 | 3-Methyladipic acid | 161.0809 | M+H | 161.0806 | 1.78 |  |
| 207 | Alanyl-alanine | 161.0921 | M+H | 161.0929 | 4.88 |  |
| 208 | Hydroxyoctanoic acid | 161.1172 | M+H | 161.117 | 1.48 |  |
|  |  | 178.1437 | M+NH4 | 178.1435 | 0.97 |  |
| 209 | beta-Homolysine | 161.1324 | M+H | 161.1323 | 0.67 |  |
| 210 | N-Methyllysine | 161.1363 | M+H | 161.1363 | 0.27 |  |
| 211 | N-Methyl-glutamic acid | 162.0761 | M+H | 162.0759 | 1.49 |  |
| 212 | Carnitine | 162.1125 | M+H | 162.1122 | 1.77 | √ |
| 213 | Caprylic acid | 162.1488 | M+NH4 | 162.1486 | 1.12 |  |
|  |  | 167.1042 | M+Na | 167.1037 | 2.71 |  |
| 214 | 4-Hydroxycoumarin | 163.0387 | M+H | 163.0387 | 0.24 | √ |
| 215 | 2,4-Hexadienyl acetate | 163.0729 | M+Na | 163.0723 | 3.68 |  |
| 216 | Hydroxylysine | 163.1077 | M+H | 163.1078 | 0.45 |  |
| 217 | Methoxy-3-heptanethiol | 163.1151 | M+H | 163.1153 | 1.27 |  |
| 218 | Octanetriol | 163.1329 | M+H | 163.1326 | 1.77 |  |
| 219 | 2-Methylamino-1-phenylbutane | 164.1432 | M+H | 164.1431 | 0.8 |  |
| 220 | 2-Hydroxy cinnamic acid | 165.0546 | M+H | 165.0546 | 0.28 | √ |
| 221 | 4-ene-Valproic acid | 165.0886 | M+Na | 165.088 | 3.57 |  |
| 222 | 2-Amino-4-hydroxy-3-methylpentanoic acid | 165.1233 | M+NH4 | 165.1238 | 2.83 |  |
| 223 | Phenylalanine | 166.0863 | M+H | 166.0861 | 1.42 | √ |
| 224 | 3-Methylglutaconic acid | 167.0315 | M+Na | 167.0311 | 2.69 |  |
| 225 | Methylxanthine | 167.0551 | M+H | 167.0552 | 0.56 |  |
| 226 | 10-Undecenoic Acid | 167.1432 | M+H-H2O | 167.1428 | 2.36 | √ |
| 227 | Norepinephrine | 170.0811 | M+H | 170.0809 | 1.42 |  |
| 228 | 2-Aminobiphenyl | 170.0961 | M+H | 170.0962 | 0.75 |  |
| 229 | 2-Isopropyl-1,4-benzenediol | 170.1175 | M+NH4 | 170.1173 | 1.36 |  |
| 230 | Glycerol 1-propanoate | 171.0628 | M+Na | 171.0624 | 2.6 |  |
| 231 | Lilac alcohol | 171.138 | M+H | 171.1376 | 2.2 |  |
| 232 | 3-Cyclohexyl-1,1-dimethylurea | 171.1492 | M+H | 171.1489 | 1.93 | √ |
| 233 | Methyl nonyl ketone | 171.1742 | M+H | 171.174 | 1.1 |  |
| 234 | 1-Acetylpiperidine-2-carboxylic acid | 172.0968 | M+H | 172.0965 | 1.82 | √ |
| 235 | Dimethyl-2,5-heptadienoic acid | 172.1332 | M+NH4 | 172.1329 | 2.03 |  |
| 236 | 1,2,2,6,6-Pentamethyl-4-piperidinol | 172.1696 | M+H | 172.1692 | 2.12 | √ |
| 237 | Hydrocinnamic acid | 173.0573 | M+Na | 173.0572 | 0.68 |  |
| 238 | Octenedioic acid | 173.0809 | M+H | 173.0807 | 1.09 |  |
|  |  | 190.1074 | M+NH4 | 190.107 | 2.06 |  |
| 239 | Glycyl-proline | 173.0924 | M+H | 173.0924 | 0.03 |  |
| 240 | Monocaproin | 173.1171 | M+H-H2O | 173.117 | 0.86 |  |
| 241 | 7-Hydroxy-3,7-dimethyloctanal | 173.1536 | M+H | 173.1533 | 1.47 |  |
| 242 | Acetyl-glutamate 5-semialdehyde | 174.0761 | M+H | 174.0759 | 1.29 |  |
| 243 | Leucine propyl ester | 174.1486 | M+H | 174.1486 | 0.16 |  |
| 244 | Dimethyl adipate | 175.0965 | M+H | 175.0962 | 1.44 |  |
|  |  | 192.123 | M+NH4 | 192.1226 | 1.92 |  |
| 245 | Arginine | 175.1149 | M+H | 175.1155 | 3.41 | √ |
| 246 | Hydroxynonanoic acid | 175.1329 | M+H | 175.1326 | 1.9 |  |
| 247 | Butyl-serine methyl ester | 176.128 | M+H | 176.1278 | 0.95 |  |
| 248 | 7-Hydroxy-4-methylcoumarin | 177.1635 | M+H | 177.1635 | 0.15 |  |
| 249 | 2-amino-3-prop-2-enylsulfinylpropanoic acid | 178.0087 | M+H | 178.0085 | 1.29 |  |
| 250 | Oxoadipic acid | 178.071 | M+NH4 | 178.0708 | 1.16 |  |
| 251 | 1,2,3,4-Tetrahydro-3-isoquinolinecarboxylic acid | 178.0863 | M+H | 178.086 | 1.79 | √ |
| 252 | Methyladipic acid | 178.1074 | M+NH4 | 178.1071 | 1.84 |  |
|  |  | 183.0628 | M+Na | 183.0625 | 1.64 |  |
| 253 | Aminoadipic acid | 179.1026 | M+NH4 | 179.1029 | 1.57 |  |
|  |  | 184.058 | M+Na | 184.0578 | 1.06 |  |
| 254 | Glucosamine | 180.0867 | M+H | 180.0864 | 1.88 |  |
| 255 | Carboxymethyl-cysteine | 180.1592 | M+H | 180.1591 | 0.47 |  |
| 256 | theobromine | 181.0835 | M+H | 181.0829 | 3.43 |  |
| 257 | Dodecalactone | 181.1587 | M+H-H2O | 181.1584 | 1.71 | √ |
| 258 | Tyrosine | 182.0804 | M+H | 182.081 | 3.33 | √ |
| 259 | 1-Pyrimidinylpiperazine | 182.14 | M+NH4 | 182.1396 | 2.07 |  |
| 260 | Methionine sulfone | 182.1651 | M+H | 182.1651 | 0.07 |  |
| 261 | Iditol | 183.0863 | M+H | 183.0855 | 4.12 |  |
| 262 | Decenedioic acid | 183.1022 | M+H-H2O | 183.1015 | 4.09 |  |
| 263 | 9-Hydroxy-10-undecenoic acid | 183.1385 | M+H-H2O | 183.1378 | 3.91 |  |
|  |  | 201.1485 | M+H | 201.1481 | 1.76 |  |
| 264 | Dodecanoic acid | 183.1749 | M+H-H2O | 183.1741 | 4.51 |  |
|  |  | 201.1849 | M+H | 201.1845 | 1.81 |  |
| 265 | Phosphorylcholine | 184.0733 | M | 184.0731 | 1.23 |  |
| 266 | Arabinonic acid | 184.0815 | M+NH4 | 184.0818 | 1.39 |  |
|  |  | 189.0369 | M+Na | 189.037 | 0.6 |  |
| 267 | Ornithino-alanine | 184.1092 | M+H-2H2O | 184.109 | 1.17 |  |
| 268 | 2-Hydroxyadipic acid | 185.042 | M+Na | 185.0418 | 0.99 |  |
| 269 | Penmacric acid | 185.0563 | M+H-H2O | 185.057 | 3.9 |  |
| 270 | Isovaleroxyisovaleric acid | 185.1178 | M+H-H2O | 185.1171 | 3.83 |  |
| 271 | Undecenoic acid | 185.1536 | M+H | 185.1533 | 1.39 |  |
| 272 | cyclododecanol | 185.1898 | M+H | 185.1897 | 0.49 |  |
| 273 | Methyl 3-phenylpropanoate | 187.0729 | M+Na | 187.0727 | 1.02 |  |
| 274 | 5-(2-Methylpropyl)tetrahydro-2-oxo-3-furancarboxylic acid | 187.0965 | M+H | 187.0962 | 1.7 |  |
|  |  | 209.0784 | M+Na | 209.0779 | 2.63 |  |
| 275 | Dihydroxyphenylglycol | 188.0917 | M+NH4 | 188.0914 | 1.52 |  |
| 276 | 7-Keto-8-aminopelargonic acid | 188.1279 | M+H | 188.1278 | 0.61 |  |
| 277 | Leucine butyl ester | 188.1644 | M+H | 188.1642 | 1.22 |  |
| 278 | N-Acetylspermidine | 188.1758 | M+H | 188.1754 | 2.01 |  |
|  |  | 210.1577 | M+Na | 210.1567 | 4.89 |  |
| 279 | Butyl-threonine methyl ester | 190.1434 | M+H | 190.1434 | 0.02 |  |
| 280 | Kynurenine | 191.0821 | M+H-H2O | 191.083 | 4.81 |  |
| 281 | alpha,epsilon-Diaminopimelic acid | 191.1041 | M+H | 191.1037 | 1.88 |  |
| 282 | Shikimic acid | 192.0866 | M+NH4 | 192.0863 | 1.37 |  |
| 283 | Diethyltoluamide | 192.1383 | M+H | 192.1379 | 1.88 | √ |
| 284 | Deoxyuridine | 193.0619 | M+H-2H2O | 193.0623 | 1.83 |  |
| 285 | N-Acetyl-aspartic acid | 193.0819 | M+NH4 | 193.0827 | 3.97 |  |
| 286 | Glucurono-6,3-lactone | 194.0659 | M+NH4 | 194.0656 | 1.57 |  |
| 287 | Ferulic Acid | 195.0649 | M+H | 195.065 | 0.34 |  |
| 288 | Tetraethylene glycol | 195.1227 | M+H | 195.1223 | 2.27 | √ |
| 289 | Geranylacetone | 195.1743 | M+H | 195.174 | 1.57 |  |
| 290 | Isoxanthopterin | 197.0781 | M+NH4 | 197.0779 | 0.77 |  |
| 291 | N-Acetyl-histidine | 198.0854 | M+H | 198.0859 | 2.77 |  |
| 292 | Azacyclotridecan-2-one | 198.1852 | M+H | 198.1849 | 1.38 | √ |
| 293 | Glycerol 1,2-diacetate | 199.0577 | M+Na | 199.0573 | 1.93 |  |
| 294 | 5-Dodecenoic acid | 199.1685 | M+H | 199.1689 | 2.09 | √ |
| 295 | Propionylcarnitine | 200.1287 | M+H-H2O | 200.1278 | 4.52 |  |
| 296 | Aminomethyldihydrolipoamide | 201.089 | M+H-2H2O | 201.0882 | 3.77 |  |
| 297 | 5-Pentyltetrahydro-2-oxo-3-furancarboxylic acid | 201.1122 | M+H | 201.1118 | 2.12 |  |
| 298 | Pantothenic acid | 202.1074 | M+H-H2O | 202.107 | 2 | √ |
|  |  | 220.118 | M+H | 220.1176 | 1.82 | √ |
| 299 | Cer 8:1;2O/2:0 | 202.1435 | M+H | 202.1434 | 0.34 |  |
| 300 | 11-aminoundecanoicacid | 202.1797 | M+H | 202.1798 | 0.44 |  |
| 301 | N,N-Dimethyldecylamine oxide | 202.2165 | M+H | 202.2161 | 2.01 | √ |
| 302 | Glucose | 203.0526 | M+Na | 203.0522 | 2.1 |  |
| 303 | Spermine | 203.223 | M+H | 203.2228 | 1.12 | √ |
| 304 | N-Acetyl-glucosamine | 204.0872 | M+H-H2O | 204.0864 | 4.01 |  |
|  |  | 239.1237 | M+NH4 | 239.1237 | 0.05 |  |
| 305 | Acetyl-carnitine | 204.123 | M+H | 204.1227 | 1.28 | √ |
| 306 | Mannitol | 205.068 | M+Na | 205.0679 | 0.66 |  |
| 307 | Val-Ser | 205.117 | M+H | 205.118 | 4.64 |  |
| 308 | 1-Phenylisoquinoline | 206.089 | M+H | 206.089 | 0.02 |  |
| 309 | Panthenol | 206.1386 | M+H | 206.1383 | 1.3 |  |
| 310 | Thymidine | 207.0776 | M+H-2H2O | 207.0779 | 1.26 |  |
| 311 | N-Acetyl-glutamic acid | 207.0975 | M+NH4 | 207.0983 | 3.68 |  |
| 312 | Chalcone | 209.0957 | M+H | 209.0957 | 0.2 |  |
| 313 | Jasmonic acid | 211.1325 | M+H | 211.1325 | 0.13 |  |
| 314 | 1-(Cyclohexylmethyl)proline | 212.1642 | M+H | 212.1641 | 0.35 |  |
| 315 | N-Ethyl-4-menthane-3-carboxamide | 212.2005 | M+H | 212.2005 | 0.1 |  |
| 316 | N-Butylbenzenesulfonamide | 214.0896 | M+H | 214.0892 | 1.97 | √ |
| 317 | N-(9-oxodecyl)acetamide | 214.1798 | M+H | 214.1797 | 0.34 |  |
| 318 | Ethyl 3-(N-butylacetamido)propionate | 216.1594 | M+H | 216.1591 | 1.43 | √ |
| 319 | Dodecenoic acid | 216.1958 | M+NH4 | 216.1955 | 1.19 |  |
| 320 | Isoferulic acid | 217.0466 | M+Na | 217.0468 | 0.89 |  |
| 321 | 3-Hydroxydodecanoic acid | 217.1798 | M+H | 217.1795 | 1.46 | √ |
| 322 | Propionyl-carnitine | 218.1393 | M+H | 218.1383 | 4.49 | √ |
| 323 | Cer 8:0;2O/3:0 | 218.1746 | M+H | 218.1748 | 0.97 |  |
| 324 | Asymmetric dimethylarginine | 220.1768 | M+NH4 | 220.177 | 0.91 |  |
| 325 | Vanillylmandelic acid | 221.042 | M+Na | 221.0418 | 0.9 |  |
| 326 | N-Acetyl-glucosaminylamine | 221.1132 | M+H | 221.1127 | 2.23 |  |
| 327 | Acetylcarnitine | 221.1496 | M+NH4 | 221.1489 | 3.04 |  |
| 328 | Ribothymidine | 223.0725 | M+H-2H2O | 223.0729 | 1.71 |  |
| 329 | Diethyl phthalate | 223.0965 | M+H | 223.0962 | 1.37 | √ |
| 330 | Cytosine | 224.0993 | M+H | 224.0995 | 0.93 |  |
| 331 | N-Acetyl-galactosaminitol | 224.1129 | M+H | 224.1125 | 1.67 | √ |
|  |  | 246.0948 | M+Na | 246.0946 | 0.99 | √ |
| 332 | 3-Hydroxy-kynurenine | 225.0907 | M+H | 225.0906 | 0.67 |  |
| 333 | Methyl jasmonate | 225.1484 | M+H | 225.1481 | 1.28 |  |
| 334 | Pentadecanoic acid | 225.2219 | M+H-H2O | 225.221 | 4.19 |  |
|  |  | 243.2319 | M+H | 243.2314 | 2.04 |  |
| 335 | Tryptophanamide | 226.097 | M+Na | 226.0972 | 1.06 |  |
| 336 | Adenosyl-methionine | 226.951 | M+H | 226.9511 | 0.36 |  |
| 337 | 1,11-Undecanedicarboxylic acid | 227.1642 | M+H-H2O | 227.1638 | 1.66 | √ |
| 338 | Myristoleic acid | 227.2006 | M+H | 227.2002 | 1.62 |  |
|  |  | 244.2271 | M+NH4 | 244.2268 | 1.17 |  |
| 339 | Leucyl-proline | 229.1431 | M+H | 229.143 | 0.44 |  |
| 340 | Myristic acid | 229.2162 | M+H | 229.2159 | 1.49 |  |
| 341 | N,N-Dimethyldodecylamine N-oxide | 230.2478 | M+H | 230.2475 | 1.31 | √ |
| 342 | Dodecanedioic acid | 231.1587 | M+H | 231.1587 | 0.04 |  |
| 343 | Acylcarnitine C4:0 | 232.1543 | M+H | 232.154 | 1.38 | √ |
| 344 | Cer 8:0;2O/4:0 | 232.1904 | M+H | 232.1905 | 0.25 |  |
| 345 | N-Succinyl-ornithine | 233.1132 | M+H | 233.1124 | 3.36 |  |
| 346 | Thr-Leu | 233.1493 | M+H | 233.1492 | 0.31 |  |
| 347 | 3-Oxo-1,8-octanedicarboxylic acid | 234.1332 | M+NH4 | 234.1332 | 0.17 |  |
| 348 | Hexadecadienoic acid | 235.2062 | M+H-H2O | 235.2052 | 4.42 |  |
| 349 | Triacetin | 236.1128 | M+NH4 | 236.1124 | 1.57 |  |
| 350 | Hydroxydodecanoate | 239.1614 | M+Na | 239.1623 | 3.77 |  |
| 351 | 2-Hydroxydecanedioic acid | 241.1046 | M+Na | 241.1043 | 1.18 |  |
| 352 | Heptadecane | 241.2521 | M+H | 241.2521 | 0.02 |  |
| 353 | Pantothenate | 242.0995 | M+Na | 242.0994 | 0.34 |  |
| 354 | Acylcarnitine C5:1 | 244.1543 | M+H | 244.1539 | 1.51 |  |
| 355 | 3-Dodecyloxypropylamine | 244.2635 | M+H | 244.2632 | 1.12 | √ |
| 356 | Uridine | 245.0768 | M+H | 245.078 | 4.99 |  |
| 357 | Diethyl azelate | 245.1742 | M+H | 245.1743 | 0.57 |  |
| 358 | 2-Methylbutyryl-carnitine | 246.17 | M+H | 246.1696 | 1.62 | √ |
| 359 | Menthone 1,3-glyceryl ketal | 246.2063 | M+NH4 | 246.206 | 1.11 |  |
| 360 | Glu-Val | 247.1288 | M+H | 247.1294 | 2.37 | √ |
| 361 | N-Succinyl-glutamic acid 5-semialdehyde | 249.1081 | M+NH4 | 249.1085 | 1.57 |  |
| 362 | Valerenolic acid | 251.1642 | M+H | 251.1635 | 2.98 |  |
| 363 | Dodecanedioic aicd | 253.141 | M+Na | 253.1406 | 1.76 |  |
| 364 | 5-Carboxy-2'-deoxyuridine | 255.0618 | M+H-H2O | 255.0625 | 2.6 |  |
| 365 | Glycerol 1-propanoate diacetate | 255.0839 | M+Na | 255.0834 | 1.78 |  |
| 366 | Palmitamide | 256.2635 | M+H | 256.2631 | 1.63 | √ |
| 367 | 9-Carboxymethoxymethylguanine | 257.0993 | M+NH4 | 257.0992 | 0.39 |  |
| 368 | Palmitic acid | 257.2475 | M+H | 257.2469 | 2.28 |  |
| 369 | Glycerophosphocholine | 258.1101 | M+H | 258.1099 | 0.71 | √ |
|  |  | 280.092 | M+Na | 280.0918 | 0.82 | √ |
| 370 | Dibutyl adipate | 259.1904 | M+H | 259.1899 | 1.79 | √ |
| 371 | Acylcarnitine C6:0 | 260.1856 | M+H | 260.1854 | 0.85 | √ |
| 372 | Glu-Leu | 261.1457 | M+H | 261.1455 | 0.58 | √ |
| 373 | 3-Hydroxyisovaleroylcarnitine | 262.1649 | M+H | 262.1646 | 1.18 | √ |
| 374 | N-(10-hydroxy-10-methylundecyl)acetamide | 266.2108 | M+Na | 266.2109 | 0.45 |  |
| 375 | 15-Methylpalmitic Acid | 271.2632 | M+H | 271.2626 | 2.23 |  |
| 376 | Lauryldiethanolamine | 274.2741 | M+H | 274.2737 | 1.61 | √ |
| 377 | Stearic acid | 285.2788 | M+H | 285.2783 | 1.86 |  |
|  |  | 307.2607 | M+Na | 307.2601 | 1.91 |  |
| 378 | Estrone | 288.1958 | M+NH4 | 288.1954 | 1.32 |  |
| 379 | Octanoylcarnitine | 288.2169 | M+H | 288.2165 | 1.3 | √ |
| 380 | C17-Sphinganine | 288.2892 | M+H | 288.2892 | 0.16 |  |
| 381 | Acylcarnitine C7-OH | 290.1964 | M+H | 290.1957 | 2.53 |  |
| 382 | Dihydrosphingosine | 302.3043 | M+H | 302.3049 | 2.09 | √ |
| 383 | Glutathione | 308.0911 | M+H | 308.0905 | 2.07 |  |
|  |  | 330.073 | M+Na | 330.0724 | 1.74 |  |
| 384 | Hexadecanedioic acid | 309.2036 | M+Na | 309.2031 | 1.63 |  |
| 385 | Gly-Tyr-Ala | 310.1397 | M+H | 310.1406 | 2.92 |  |
| 386 | 7,8-Dihydropteroic acid | 315.12 | M+H | 315.1197 | 1.05 |  |
| 387 | SPB 19:0;2O | 316.3205 | M+H | 316.3205 | 0.05 |  |
| 388 | alpha-CEHC | 317.115 | M+K | 317.1144 | 1.97 |  |
| 389 | N-Benzyloleamide | 372.3188 | M+H | 372.3182 | 1.68 |  |
| 390 | DG O-13:1_6:0 | 388.3414 | M+NH4 | 388.3413 | 0.23 |  |

**Table S3.** Clinical information of lung cancer patients in the training cohort.

| **Patient no.** | **Sex** | **Age** | **Stage** | **Total CTC number** |
| --- | --- | --- | --- | --- |
| 1 | Female | 68 | stage Ⅳ cT4N1M1 | 4 |
| 2 | Female | 36 | stage Ⅳ cT4N3M1 | 3 |
| 3 | Male | 59 | stage Ⅳ cT3N4M1 | 4 |
| 4 | Male | 76 | stage Ⅳ cT3N2M1 | 11 |
| 5 | Female | 46 | stage Ⅳ cT4N3M1 | 4 |
| 6 | Female | 67 | stage Ⅳ cT4N2M1 | 3 |
| 7 | Female | 63 | stage Ⅳ cT1N2M1 | 3 |
| 8 | Female | 42 | stage Ⅳ cT4N2M1 | 5 |
| 9 | Male | 58 | stage Ⅳ cT3N2M1 | 5 |
| 10 | Female | 52 | stage Ⅳ cT4N1M1 | 3 |
| 11 | Female | 70 | stage Ⅳ cT3N3M1 | 5 |
| 12 | Male | 58 | stage Ⅳ cT4N3M1 | 2 |
| 13 | Female | 72 | stage Ⅳ cT2N2M1 | 4 |
| 14 | Female | 56 | stage Ⅳ cT2N3M1 | 5 |
| 15 | Female | 55 | stage Ⅳ cT1bN0M1 | 3 |
| 16 | Male | 48 | stage Ⅳ cT4N2M1 | 4 |
| 17 | Female | 63 | stage Ⅳ cT1cN2M1 | 4 |
| 18 | Male | 55 | stage Ⅳ cT4N3M1 | 5 |
| 19 | Male | 56 | stage Ⅳ cT4N3M1 | 7 |
| 20 | Male | 80 | stage Ⅳ cT4N3M1 | 3 |
| 21 | Male | 67 | stage Ⅲb cT2bN3M0 | 3 |
| 22 | Male | 64 | stage Ⅲb cT2N2M0 | 4 |
| 23 | Male | 74 | stage Ⅲc cT4N3M0 | 2 |
| 24 | Male | 74 | stage Ⅲa cT2N0M0 | 4 |
| 25 | Female | 51 | stage Ⅲa cT1N2M0 | 5 |
| 26 | Male | 67 | stage Ⅲb cT4N2M0 | 3 |
| 27 | Female | 65 | stage Ⅲa cT1cN2M0 | 3 |
| 28 | Male | 68 | stage Ⅲb cT3N2M0 | 3 |
| 29 | Female | 75 | stage Ⅲb cT1N3M0 | 4 |
| 30 | Female | 70 | stage Ⅲc cT4N3M0 | 2 |

**Table S4.** Clinical characteristics and biomarker levels of lung cancer patients in the training cohort. NSE neuron-specific enolase, CEA carcinoembryonic antigen, CA 125 carbohydrate antigen 125, CYFRA21-1 Serum keratin 19.

|  | **Training cohort (*n* =30)** |
| --- | --- |
| Sex |  |
| male | 14 (46.7%) |
| female | 16 (53.3%) |
| Age (years) |  |
| median | 63.5 |
| range | 36-80 |
| Tumor |  |
| T1 | 6 (20.0%) |
| T2 | 5 (16.7%) |
| T3 | 5 (16.7%) |
| T4 | 14 (46.7%) |
| NSE (ng/ml) |  |
| <16.3 | 13 (43.3%) |
| ≥16.3 | 17 (56.7%) |
| CEA (ng/ml) |  |
| <5 | 14 (46.7%) |
| ≥5 | 16 (53.3%) |
| CA125 (u/ml) |  |
| <30.2 | 23 (76.7%) |
| ≥30.2 | 7 (23.3%) |
| CYFRA21-1 (ng/ml) |  |
| <3.3 | 16 (53.3%) |
| ≥3.3 | 14 (46.7%) |

**Table S5.** Total CTC count, CTC1 count, CTC2 count and CTC3 count of each patient in the training cohort.

| **Patient.**  **ID** | **Distant metastasis status** | **Total CTC**  **Count** | **Classified CTC count** | | |
| --- | --- | --- | --- | --- | --- |
|  |  |  | **CTC1 subgroup** | **CTC2 subgroup** | **CTC3 subgroup** |
| 1 | Brain | 4 | 4 | 0 | 0 |
| 2 | Brain | 3 | 1 | 0 | 2 |
| 3 | Brain | 4 | 0 | 3 | 1 |
| 4 | Brain | 11 | 7 | 0 | 4 |
| 5 | Brain | 4 | 2 | 1 | 1 |
| 6 | Brain | 3 | 1 | 1 | 1 |
| 7 | Brain | 3 | 3 | 0 | 0 |
| 8 | Brain | 5 | 4 | 0 | 1 |
| 9 | Brain | 5 | 4 | 0 | 1 |
| 10 | Brain | 3 | 0 | 3 | 0 |
| 11 | Bone | 5 | 1 | 2 | 2 |
| 12 | Bone | 2 | 0 | 2 | 0 |
| 13 | Bone | 4 | 0 | 3 | 1 |
| 14 | Bone | 5 | 1 | 3 | 1 |
| 15 | Bone | 3 | 0 | 3 | 0 |
| 16 | Bone | 4 | 0 | 3 | 1 |
| 17 | Bone | 4 | 0 | 2 | 2 |
| 18 | Bone | 5 | 0 | 4 | 1 |
| 19 | Bone | 7 | 2 | 3 | 2 |
| 20 | Bone | 3 | 1 | 2 | 0 |
| 21 | Non. | 3 | 3 | 0 | 0 |
| 22 | Non. | 4 | 2 | 0 | 2 |
| 23 | Non. | 2 | 0 | 1 | 1 |
| 24 | Non. | 4 | 1 | 1 | 2 |
| 25 | Non. | 5 | 3 | 0 | 2 |
| 26 | Non. | 3 | 1 | 2 | 0 |
| 27 | Non. | 3 | 1 | 0 | 2 |
| 28 | Non. | 3 | 1 | 1 | 1 |
| 29 | Non. | 4 | 0 | 2 | 2 |
| 30 | Non. | 2 | 1 | 0 | 1 |

**Table S6.** Proportions of CTCs across different patient cohorts within each cluster.

| **CTC Proportions/%** | **Bone**  **Metastasis** | **Brain Metastasis** | **Non-metastatic** | **Total** |
| --- | --- | --- | --- | --- |
| CTC1 | 11.4 | 59.1 | 29.5 | 100.0 |
| CTC2 | 64.3 | 19.0 | 16.7 | 100.0 |
| CTC3 | 29.4 | 32.4 | 38.2 | 100.0 |

**Table S7.** Univariate logistic regression analyses of CTC counts, clinical characteristics and biomarker levels with brain metastatic risk of lung cancer in the training cohort. Statistical analysis was performed using the logistic regression models. NSE neuron-specific enolase, CEA carcinoembryonic antigen, CA 125 carbohydrate antigen 125, CYFRA21-1 Serum keratin 19.

|  | **Non-brain metastasis** | **Brain metastasis** | ***p*** |
| --- | --- | --- | --- |
| Sex |  |  | 0.203 |
| male | 3 | 11 |  |
| female | 7 | 9 |  |
| Age (years) |  |  | 0.075 |
| < 60 | 6 | 7 |  |
| ≥ 60 | 4 | 13 |  |
| NSE (ng/ml) |  |  | 0.397 |
| < 16.3 | 4 | 9 |  |
| ≥ 16.3 | 6 | 11 |  |
| CEA (ng/ml) |  |  | 0.407 |
| < 5 | 6 | 8 |  |
| ≥ 5 | 4 | 12 |  |
| CA125 (u/ml) |  |  | 0.483 |
| < 30.2 | 8 | 15 |  |
| ≥ 30.2 | 2 | 5 |  |
| CYFRA21-1 (ng/ml) |  |  | 0.2 |
| < 3.3 | 5 | 11 |  |
| ≥ 3.3 | 5 | 9 |  |
| Total CTC Count |  |  | 0.289 |
| ≤ 3 | 4 | 9 |  |
| > 3 | 6 | 11 |  |
| CTC1 Count |  |  | 0.023 |
| ≤ 1 | 4 | 16 |  |
| > 1 | 6 | 4 |  |
| CTC2 Count |  |  | 0.082 |
| ≤ 1 | 8 | 8 |  |
| > 1 | 2 | 12 |  |
| CTC3 Count |  |  | 0.889 |
| ≤ 1 | 8 | 12 |  |
| > 1 | 2 | 8 |  |

**Table S8.** Univariate and multivariate logistic regression analyses of CTC counts, clinical characteristics and biomarker levels with bone metastatic risk of lung cancer in the training cohort. Statistical analysis was performed using the logistic regression models. NSE neuron-specific enolase, CEA carcinoembryonic antigen, CA 125 carbohydrate antigen 125, CYFRA21-1 Serum keratin 19.

|  | **Non-bone metastasis** | **Bone metastasis** | ***p*** | |
| --- | --- | --- | --- | --- |
|  |  |  | **Univariate** | **Multivariate** |
| Sex |  |  | 0.796 |  |
| male | 5 | 9 |  |  |
| female | 5 | 11 |  |  |
| Age (years) |  |  | 0.845 |  |
| <60 | 6 | 7 |  |  |
| ≥60 | 4 | 13 |  |  |
| NSE (ng/ml) |  |  | 0.702 |  |
| <16.3 | 3 | 10 |  |  |
| ≥16.3 | 7 | 10 |  |  |
| CEA (ng/ml) |  |  | 0.631 |  |
| <5 | 2 | 12 |  |  |
| ≥5 | 8 | 8 |  |  |
| CA125 (u/ml) |  |  | 0.665 |  |
| <30.2 | 7 | 16 |  |  |
| ≥30.2 | 3 | 4 |  |  |
| CYFRA21-1 (ng/ml) |  |  | 0.794 |  |
| <3.3 | 6 | 10 |  |  |
| ≥3.3 | 4 | 10 |  |  |
| Total CTC Count |  |  | 0.651 |  |
| ≤3 | 3 | 10 |  |  |
| >3 | 7 | 10 |  |  |
| CTC1 Count |  |  | 0.044 | 0.85 |
| ≤1 | 9 | 11 |  |  |
| >1 | 1 | 9 |  |  |
| CTC2 Count |  |  | 0.004736 | 0.014 |
| ≤1 | 0 | 16 |  |  |
| >1 | 10 | 4 |  |  |
| CTC3 Count |  |  | 0.577 |  |
| ≤1 | 7 | 13 |  |  |
| >1 | 3 | 7 |  |  |
